# Low intensity ultrasound-mediated drug-loaded nanoparticles intravaginal drug delivery: an effective synergistic therapy scheme for treatment of vulvovaginal candidiasis

**DOI:** 10.1186/s12951-023-01800-x

**Published:** 2023-02-13

**Authors:** Min Yang, Yuchao Cao, Zhifei Zhang, Jiajun Guo, Can Hu, Zhibiao Wang, Yonghong Du

**Affiliations:** 1 State Key Laboratory of Ultrasound in Medicine and Engineering, College of Biomedical Engineering, Chongqing Medical University, No. 1 Yixueyuan Road, Yuzhong District, Chongqing, 400016 China; 2 Chongqing Key Laboratory of Biomedical Engineering, Chongqing Medical University, Chongqing, 400016 China

**Keywords:** Vulvovaginal candidiasis, Low intensity ultrasound, Amphotericin B-loaded PLGA-PEG nanoparticles, Intravaginal drug delivery, Synergistic therapy

## Abstract

**Purpose:**

Vulvovaginal candidiasis (VVC) is a mucosal infection of the female lower genital tract for which treatment using conventional antifungal drugs shows limited effectiveness. Herein, amphotericin B-loaded poly(lactic-co-glycolic acid)-polyethylene glycol (PLGA-PEG) nanoparticles (AmB-NPs) were fabricated and combined with low intensity ultrasound (US) to mediate AmB-NPs intravaginal drug delivery to achieve productive synergistic antifungal activity in a rabbit model of VVC.

**Methods:**

Polymeric AmB-NPs were fabricated by a double emulsion method and the physical characteristics and biosafety of nanoparticles were analyzed. The distribution and tissue permeability of nanoparticles after intravaginal ultrasound irradiation (1.0 MHz, 1.0 W/cm^2^, 5 min, 50% duty ratio) were observed in the vagina. The synergistic therapeutic activity of US-mediated AmB-NPs treatment was evaluated using an experimental rabbit model of VVC. Vaginal *C. albicans* colony counts, the pathological structure of the vagina epithelium, and Th1/Th2/Th17-type cytokine and oxidative stress levels were analyzed to investigate the therapeutic effect in vivo.

**Results:**

The prepared AmB-NPs showed an obvious shell and core structure with uniform size and good dispersion and displayed high biosafety and US-sensitive slow drug release. Ultrasound significantly enhanced nanoparticle transport through the mucus and promoted permeability in the vaginal tissue. US-mediated AmB-NPs treatment effectively increased drug sensitivity, even in the presence of the vaginal mucus barrier in vitro. On the seventh day after treatment in vivo, the combination treatment of AmB-NPs and US significantly reduced the fungal load in the vagina, achieving over 95% clearance rates, and also improved the pathological epithelium structural damage and glycogen secretion function. The expression of Th1 (IFN-γ, IL-2) and Th17 (IL-17) cytokines were significantly increased and Th2 (IL-6, IL-10) cytokines significantly decreased in the US + AmB-NP group. Furthermore, US-mediated AmB-NPs treatment effectively increased *C. albicans* intracellular reactive oxygen species (ROS) levels and promoted vaginal oxidation and antioxidants to normal levels.

**Conclusion:**

US-mediated drug-loaded nanoparticles with intravaginal drug delivery exhibited a productive synergistic antifungal effect, which may provide a new non-invasive, safe, and effective therapy for acute or recurrent fungal vaginitis.

**Graphical Abstract:**

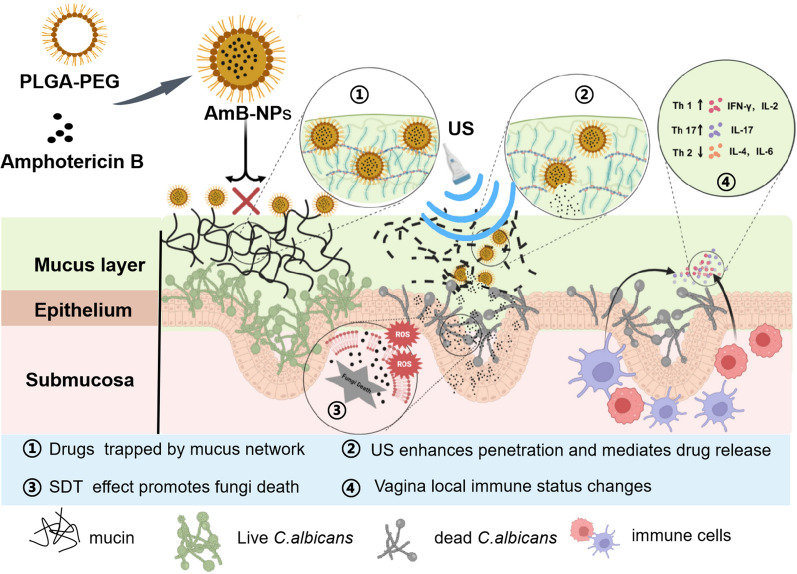

**Supplementary Information:**

The online version contains supplementary material available at 10.1186/s12951-023-01800-x.

## Introduction

Vulvovaginal candidiasis (VVC), considered as the second most common type of vaginitis infection after bacterial vaginitis, is a mucosal infection of the female lower genital tract caused by *Candida* infection, which is mainly caused by the polymorphic opportunistic pathogenic fungus *Candida albicans (C. albicans)* in 85–90% of diagnosed cases [1]. It is estimated that about 70–75% of women worldwide have symptomatic VVC infection at least once in their lifetime and 5–10% of women will be diagnosed with recurrent vaginal candidiasis (RVVC), defined as at least four episodes in one year. Early, effective, and thorough treatment is crucial to prevent recurrence and drug resistance of *C. albicans* infection [2].

In the typical symptoms of VVC patients, in addition to intense itching, redness, and swelling, an obvious increase of vaginal discharge mucus is an important clinical manifestation [3]. Normally, vaginal mucus helps to protect mucous surfaces from infection and injury, but pathogens such as *C. albicans* are able to penetrate the mucus, colonize the vaginal epithelium, and harm the submucosal tissue [4]. The cervicovaginal mucus (CVM) secretion consists of primarily linear, glycosylated mucin fibers that entangle into a dense network, which is a physical diffusion barrier that can limit drug penetration by trapping and removing foreign objects or particles, especially those with cationic or hydrophobic properties [5, 6].

The recommended standard therapy for vaginal *C. albicans* infections is based on oral or intravaginal administration of antifungal drugs, consisting of azoles, polyenes, echinocandins, acrylamides, and nucleoside analogs [7]. However, the fungal clearance and clinical effectiveness of antifungal drug therapy is limited, especially for recurrent and drug-resistant infection. For example, the triazoles (fluconazole or itraconazole) must be administered for periods spanning months to a year to guarantee the elimination of the fungus [8]. An analysis of the reasons for the poor curative effect of traditional vaginal administration revealed the following factors: insufficient diffusion of drugs on the vaginal surface; limited contact between drugs and the diseased parts of vaginal folds; and low capacity to eliminate hyphae from deep layers of the vaginal tissue, all of which are associated with vaginal mucus trapping of drugs and blocking their deep penetration in the vagina.

To overcome the deficiency of the conventional pharmaceutical dosage forms in the treatment of the VVC, the development of novel pharmaceutical dosage forms and vaginal drug delivery schemes is required. Nanoparticle-based drug delivery systems improve drug dispersion and absorption, provide a sustained release, reduce dose and drug toxicity, and exhibit the anticipated application potential in the treatment and prevention of vagina infections and even genital tract cancer [9]. Amidst the various polymers synthesized for formulating polymeric nanoparticles, poly(lactic-co-glycolic acid) (PLGA) has brought considerable attention. PLGA is one of the most successfully used biodegradable polymers because it possess many desirable properties, such as a controlled and sustained release, low cytotoxicity, biocompatibility with tissues and cells, prolonged residence time, and targeted delivery [10]. However, because of the length-scale and shear-dependent rheological and barrier properties of the CVM, the conventional polymeric PLGA nanoparticles are easily trapped and immobilized by the CVM, which makes it difficult for them to access the mucosal surface and remove submucosal pathogenic bacteria [11, 12]. A hydrophilic and uncharged molecule of poly(ethylene glycol) (PEG) covering the surface of nanoparticles effectively minimized adhesive interactions between the nanoparticles and anionic glycans or periodic hydrophobic-naked protein domains on mucins, and may be a potential strategy to improve the limitations of nanoparticles in mucus delivery [13].

Low intensity ultrasound (US), as a non-invasive, efficient, targetable, and controllable physical technique, has been successfully used to greatly enhance the permeability of the skin and blood–brain barrier, and to increase the distribution of drugs in solid tissues, which is known as the sonophoresis phenomenon [14]. Previous studies by our group confirmed that US-mediated nanoparticles delivery created many holes in the extracellular matrix of *C.albicans* thalli and biofilms, which provided a beneficial environment for the drug to enter the biofilm for sterilization [15, 16]^.^ Furthermore, the high shear force formed by the shock wave and micro-jets generated by the vibration, compression, and collapse of microbubbles in the ultrasonic cavitation process may be helpful for improving the permeability of the CVM, because under high shear stress, mucus viscosity reduces to that of water, but at low shear rates, mucus behaves like an elastic solid (1000–10,000 times more viscous than water) [17].

Amphotericin B (AmB), as a "gold standard" for invasive fungal infection treatment, is administered mainly by intravenous injection due to poor aqueous solubility, which precludes its vaginal topical delivery. Besides, acute hepatic and renal toxicity limits its dose (less than 1.5 mg/kg/day) and long-term use, especially for immunodeficient patients [18, 19]. In addition, AmB as a sonosensitizer molecule, can be activated by ultrasound to generate toxic compounds of reactive oxygen species (ROS) with consequent cell apoptosis [20].

**Fig.1:**
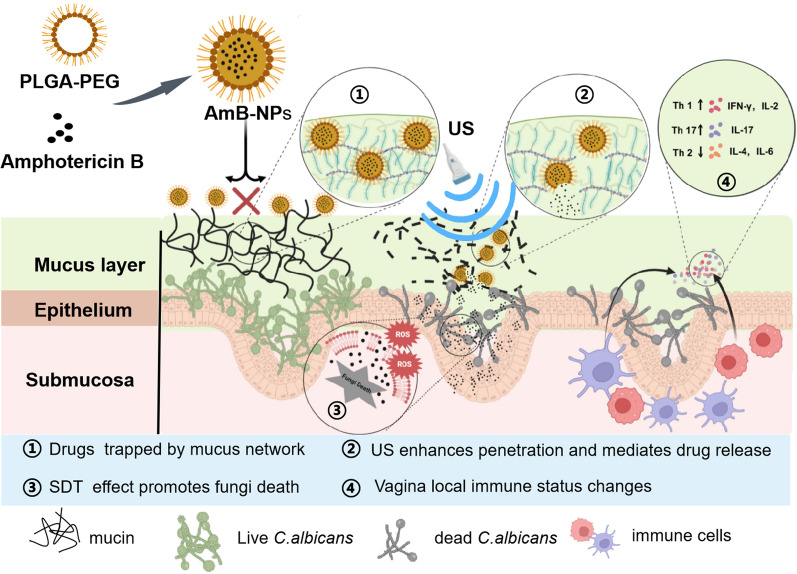
The schematic illustration of ultrasound-mediated AmB-loaded PLGA-PEG nanoparticles intravaginal synergistic therapy for vulvovaginal candidiasis

In this study, we employed amphotericin B-loaded PLGA nanoparticles coated with low molecular weight PEG for efficient drug penetration and uniform drug distribution. Then, intravaginal low-intensity ultrasound was used to further improve the permeability through the vaginal mucus barrier and mediate the controlled release of the nanoparticles. The objective of this study was to utilize US-mediated AmB-loaded PLGA-PEG nanoparticles intravaginal drug delivery to achieve a productive synergistic antifungal activity in a rabbit model of vulvovaginal candidiasis, which may provide a new non-invasive, safe, and effective therapy for acute or recurrent fungal vaginitis. The schematic illustration of US-mediated AmB-NPs intravaginal synergistic therapy is shown in Fig. 1.

## Materials and methods

### Materials and reagents

Poly(lactic-co-glycolic) acid (PLGA) (MW 15,000, ratio of lactide to glycolic acid molar ratio of 50:50), polyethylene glycol (PEG) (MW:3500), PLGA-PEG polymer material was customized from Jinan Daigang Biotechnology Co., LTD. Amphotericin B (AmB) powder (99.8% purity), crystal violet (CV),polyvinyl alcohol (PVA), dimethyl sulfoxide (DMSO), 2,3-bis(2-methoxy-4-nitro-5-sulfo-phenyl)-2H-tetrazolium-5-carboxanilide (XTT), estradiol cypionate, 1,1'-dioctadecyl-3,3,3',3'-tetramethylindo carbocyanine perchlorate (DiI) red fluorescence probe, 4,6-diamidino-2-phenylindole (DAPI), dialysis bags (molecular weight cut-off of 12 kDa) were purchased form Sigma-Aldrich (St. Louis, MO, USA). Tissue-Tek OCT Compound (Sakura Finetek U.S.A. Inc.). Sabouraud Dextrose Agar (SDA), Sabouraud Dextrose broth (SD) and Man Rogosa and Sharpe broth/Agar (MRS)were obtained from Huankai Microbial Co.(Guangdong, China). RPMI 1640 medium, Dulbecco’s Modifed Eagle’s Medium (DMEM), fetal bovine serum (FBS) and phosphate-buffered saline (PBS, pH = 7.0/5.5) was supplied by Thermo Fisher Scientific (Massachusetts, USA). ELISA kits for IL-2, IL-4, IL-6, IL-17, TNF-α and IFN-γ were purchased from Jingmei Biotechnology Co. (Jiangsu, China). CCK-8 (Dojindo, Japan).

### Microbial strains and cell culture

The *C. albicans* strain (ATCC-10231) was obtained from the China General Microbiological Culture Collection Center. A cryopreservation fungal solution was inoculated into 100 mL of SD broth at 37 °C for 24 h with agitation at 150 rpm, and the fresh *C. albicans* suspension was collected and suspended in RPMI 1640 medium containing 10% FBS and adjusted to 3.0 × 10^8^ CFU/mL for future experiments.

*Lactobacillus crispatus (L. crispatus)* (BNCC135057) was obtained from BeNa Biotechnology Co. (Hebei, China) and routinely cultured in a micro-aerobic environment at 37 °C in MRS broth overnight. The bacteria were collected and resuspended in PBS at 3.0 × 10^7^ CFU/mL. Macrophages (RAW264.7) were obtained from the Shanghai Institute of Cell Research, Chinese Academy of Sciences. The cells were cultured in DMEM containing 10% FBS and 1% penicillin–streptomycin solution at 37 °C in a 5% CO_2_ incubator. Cell growth was observed daily with an inverted microscope, and cells in logarithmic growth phase were used for experiments.

### Animals

Healthy female New Zealand rabbits, 3–4 months old, weighing 2.0–2.5 kg, were purchased from the Experimental Animal Center of Chongqing Medical University. This study complied with the ethical standards established by the Experimental Animal Ethics Committee of Chongqing Medical University (Ethical Number: 2022162).

### Drug-loaded nanoparticles preparation, characterization, and drug release

Nanoparticles were prepared using a double emulsification method [16, 21]. Briefly, AmB powder was first completely dissolved in DMSO and then diluted it with deionized water to a final concentration of 5 mg/mL as the aqueous phase and 40 mg PLGA-PEG polymer material was completely dissolved in 2 mL CHCl_3_ solution as the organic phase. Then 400 μL of AmB solution was added to 2 mL CHCl_3_ solution and the mixture was first emulsified in an ice bath by using an ultrasonic probe (XL2020 Acoustic Vibrograph, USA) at a power of 150 W (50% duty ratio) for 2 min (power 5 s, interval 5 s). Subsequently, the initial water-in-oil emulsion was added to 4 mL of a PVA solution (4% w/v) and underwent a second emulsification for another 5 min to form a water-in-oil-in-water nanoemulsion formulation. Then, 6 mL of isopropanol was added to the final solution and magnetically stirred overnight at room temperature to stabilize the NPs and volatilize the CHCl_3_. The prepared nanoparticles were stored by freeze-drying. In addition, DiI-loaded PLGA-PEG nanoparticles (DiI-NPs) and blank PLGA-PEG nanoparticles (Blank-NPs) were prepared using the above method, except that the AmB drug was exchanged for 2 mg of DiI fluorescence dye or an equal amount of deionized water.

The average diameter (AD), zeta potential (ZP) and polydispersity index (PDI) were measured by dynamic light scattering (DLS, Malvern Instruments, UK). The surface morphology and microstructure were observed by scanning electron microscopy (SEM, Hitachi S-3400N, Japan) and transmission electron microscopy (TEM, Hitachi H-7600, Japan). To determine the drug loading content (LC%) and the encapsulation efficiency (EE%) of the AmB-NPs, the drug AmB standard curve was first calculated by using a UV–vis spectrophotometer (Y = 0.1212X + 0.0129, R^2^ = 0.9976, where Y is the absorbance value at 350 nm, and X is the AmB concentration). Then, the prepared freeze-dried nanoparticles were completely dissolved in DMSO and the absorbance value of the solution at 350 nm was measured to convert the concentration of AmB, and the LC% and EE% were calculated using the following equations (a):

$${\text{LC }}\left( {\% {\text{ w}}/{\text{w}}} \right)\, = \,\left[ {{\text{Mass of drug in NPs}}/{\text{Mass of loaded NPs recovered}}} \right]\, \times \,{1}00\% ,$$

and

$${\text{EE }}\left( {\% {\text{ w}}/{\text{w}}} \right)\, = \,\left[ {{\text{Mass of drug in NPs}}/{\text{Amount of drug used in encapsulation}}} \right]\, \times \,{1}00\% .$$

The vaginal pH of a vaginitis infection is about 5.0–6.5 [22]. To more accurately reflect the release of the AmB-NPs during transvaginal therapy, the drug kinetic release in different pH conditions with low intensity ultrasound stimulation were assessed via the dialysis method in vitro [16]. Briefly, 10 mg AmB-NPs were dissolved in 10 mL PBS (pH 5.5 or 7.2) containing 5% v/v of DMSO and then exposed to ultrasonic irradiation (1.0 MHz, 1.0 W/cm^2^, 5 min, 50% duty ratio). The solutions were transferred into different dialysis bags and dialyzed against 50 mL of PBS/DMSO (95%/5% v/v) at a speed of 100 rpm. At pre-determined time points (0, 1, 2, 4, 8, 12, 24, 48, 72, 96, and 120 h), aliquots of 500 μL of the released medium were withdrawn for analysis of the drug release rate with a UV–vis spectrophotometer (UV-2600 SHIMADZU, Japan), and were then replaced with equivalent fresh PBS/DMSO (95%:5% v/v) to maintain a constant total volume of the dialysate. The cumulative amount of AmB released from the nanoparticles was calculated using the equation (b): Cumulative release (%) = [weight of AmB released from NPs/ initial weight of the drug in NPs] × 100%.

### Cytotoxicity and bio-safety analysis of the AmB-NPs in vitro and in vivo

The cytotoxicity of the AmB-NPs was analyzed using macrophages, as they are innate immune cells of the vaginal and cervical mucosa. RAW264.7 macrophages were plated in advance on 96-well culture plates (1 × 10^4^ cells/well) and incubated for 12 h. Sterilized AmB-NPs and free AmB were resuspended in serum-free DMEM at varying equivalent AmB concentrations (1.0, 2.0, 4.0, 8.0, 16.0 μg/mL) and added to the 96-well culture plates. Wells with the same amount of serum-free medium were used as the control group. After 24 h of reaction, 10% CCK-8 reagent was added into each well for another 2 h, and the activity of macrophages was detected by a Multifunctional Enzyme Label instrument (Thermo Fisher Scientific, USA) based on the absorbance at 450 nm. Furthermore, we evaluated the cytotoxicity of the AmB-NPs on *Lactobacillus crispatus*, which are dominant bacteria in the vagina of healthy women. The resuspended *L. crispatus* solution was co-cultured with sterilized AmB-NPs and free AmB at varying equivalent AmB concentrations (1.0, 2.0, 4.0, 8.0, and 16.0 μg/mL) for 24 h at 37 °C. Then the activity of bacteria was detected by an XTT assay at 490 nm. The most significant adverse effects of free AmB, nephrotoxicity, was investigated as a standard biosafety test in vivo. Healthy female New Zealand rabbits were injected with sterilized AmB-NPs and free AmB at 5 mg/kg/day for 3 days (equivalent to a fivefold clinical dose) intravenously or intravaginally perfused with a syringe. Blood serum was collected at 3 days after injection for biochemical analysis of renal function, including urea nitrogen (BUN) and creatine (SCr), using a biochemical autoanalyzer (Cobas 701, Roche).

### Selection of ultrasonic irradiation parameters and bio-safety verification

An annular ultrasonic vaginitis therapeutic instrument was equipped with a tubular annular transducer with a diameter of 1.0 cm, and a circular planar transducer with a diameter of 3.0 cm, developed by Chongqing Ronghai Engineering Research Center of Ultrasound Medicine Co., Ltd., China. Except for the ultrasonic emission mode and the shape of the transducer, the other parameters of the two transducers were completely consistent, with a center frequency of 1.0 MHz, an adjustable sound intensity output of 0–3.0 W/cm^2^, and 50% duty cycle in pulsed mode.

The freshly grown macrophages and *L. crispatus* with high activity were inoculated in 35-mm diameter culture plates and immediately subjected to ultrasonic irradiation by the circular planar transducer at an intensity of 0.5 W/cm^2^ or 1.0 W/cm^2^, and a time of 5 min, 10 min, or 15 min in a 50% duty cycle in pulsed mode. The irradiation mode was direct contact of the ultrasonic transducer with the bottom of the culture plates coated with ultrasonic coupling agent. Then, we further verified the safety of the ultrasound combined with nanoparticles. The sterilized AmB-NPs and free AmB at final equivalent AmB concentrations of 4.0 μg/mL were added to the cells and irradiated immediately at the selected intensity of 1.0 W/cm^2^ for 5 min. After ultrasonic irradiation, the culture plates were placed in the incubator for another 24 h to detect activity changes in the macrophages and bacteria using CCK-8 and XTT assays, respectively, as described previously.

Next, we verified the safety of US alone or combined with nanoparticles intravaginal irradiation in vivo. The AmB-NPs or 0.9% saline solution was injected into the rabbit vagina with a syringe at an AmB concentration of 1 mg/mL, and then the annular treatment transducer was extended into the rabbit vagina for ultrasonic irradiation at an intensity of 0.5 W/cm^2^ or 1.0 W/cm^2^ and a time of 5 min or 10 min at a 50% duty cycle in pulsed mode. Then, 24 h after irradiation, the rabbits were euthanized and the vaginal tissues were isolated, sliced, and hematoxylin and eosin (H&E) stained to observe the pathological changes.

### Ex vivo antifungal susceptibility tests of AmB and AmB-NPs

The antifungal activity of US-mediated AmB and AmB-NPs in vitro, with and without a mucus barrier, were analyzed by minimum inhibitory concentration (MIC) detection based on the National Committee for Clinical Laboratory Standard “NCCLS document M27-A3, (Standards, 2009)” [23]. Briefly, *C. albicans* cell suspensions of 3 × 10^5^ cells/mL were diluted 1:50 in RPMI 1640 medium (pH 5.5 and pH 7.2) and transferred to 24-well plates, and 500 μL of simulated vaginal mucus [24] was added to the wells covered with *C. albicans*. Then, serially diluted concentrations of AmB or AmB-NPs (0–16 μg/mL) were placed on top of the mucus layer, followed by ultrasonic irradiation at the bottom of the culture plate. The wells containing drug-free RPMI 1640 medium and inoculum were used as positive controls. After incubation at 37 °C for 24 h, the absorbance value of the wells was measured. An 80% decrease in the absorbance value compared with the positive control was the MIC of the compound, corresponding to a score of 0 in the NCCLS M27-A3 protocol.

### Investigation of the permeability of US-mediated nanoparticles delivery in mucus and vaginal tissue

The sterile DiI-NPs (1 mg/mL) mixed with the simulated vaginal mucus were injected into female rabbit vaginas with a syringe and then immediately irradiated by the tubular annular US transducer at an intensity of 1.0 W/cm^2^ for 5 min or 10 min. The control group received sham irradiation (no energy output from the ultrasonic transducer). The entire vagina was then removed after 2 h and frozen in Tissue-Tek OCT compound. Transverse sections of vaginal tissue at a thickness of 8 μm were obtained with a Microm HM 500 M cryostat (Microm International). The sections were then stained with DAPI to visualize the cell nuclei and mark the vaginal tissue outline. The distribution of the DiI-NPs (red fluorescence) in the vagina was observed by laser scanning confocal microscope (LSCM, Nikon, Japan) and the average area of red fluorescence intensity in the vaginal cavity and mucosal area was calculated by five randomly selected view fields using ImageJ software.

To further explore whether US-mediated nanoparticles affected the permeability of vaginal epithelium tissue, the rabbits were euthanized and the vaginal tissue was isolated and pruned for redundant organization. The vagina was dissected longitudinally, flattened, and cut into 1 cm × 1 cm small pieces. Then, 50 μL of DiI-NPs was added to the isolated vaginal mucosal surface, followed by ultrasonic irradiation treatment. After 2 h, the remaining non-permeable nanoparticles on the mucosal surface were gently removed with PBS. The penetration depth and distribution of nanoparticles in the vaginal tissue were observed longitudinally by LSCM scanning and the average area of red fluorescence intensity in the mucosa and submucosa layers was calculated by five randomly selected view fields (the mucosal layer thickness of about 60 μm was used as the dividing line).

### Establishment of the VVC model in rabbits

The experimental vulvovaginal candidiasis model in rabbits was established by an improved fungal fluid intravaginal perfusion method according to Song [25]. Prior to infection, animals were subcutaneously injected with 1.2 mg/kg estradiol valerate once a day for three consecutive days prior to inoculation to induce false estrus conditions and an immunosuppressed environment. Then, *C. albicans* RPMI 1640 (10% FBS) suspensions (100 μL/rabbit, containing 3 × 10^8^ CFU/mL) were inoculated intravaginally once a day for five consecutive days using an automatic micropipette, and the vaginal opening was blocked with aseptic cotton balls to prevent the outflow of fluid. After 5 days of consecutive inoculation, the vulva redness, swelling, and secretions were observed, and Gram staining was performed on vaginal swabs. A vaginal tissue biopsy was performed and H&E staining of the tissue was observed by light microscopy. In addition, vaginal lavage fluid of each animal was collected and cultured on SDA to count the number of yeast cells during the vaginal infection.

### US-mediated nanoparticles therapy in vivo

The successfully modeled rabbits were randomly assigned into six groups (n = 5) and treated as follows: control (infected animals treated with 0.2 mL of sterile saline solution); US (infected animals treated with 0.2 mL of sterile saline solution and US irradiation); AmB (infected animals treated with 0.2 mL of a pure AmB solution of 1.0 mg/mL); AmB + US (infected animals treated with 0.2 mL of a pure AmB solution of 1.0 mg/mL and US irradiation); AmB-NPs (infected animals treated with 0.2 mL of an AmB-NPs solution of 20 mg/mL, equivalent to a pure AmB concentration of 1.0 mg/mL according to the drug loading rate); and AmB-NPs + US (infected animals treated with 0.2 mL of an AmB-NPs solution of 20 mg/mL and US irradiation). The US-treatment animals were irradiated intravaginal by a tubular annular transducer immediately after drug or saline injection at an intensity of 1.0 W/cm^2^ for 5 min with a 50% duty cycle in pulsed mode. All treatments were performed three consecutive times at intervals of one day. The timeline of the experimental animal treatment is shown in Fig. 5A.

### Inflammation score and CFU analysis

After the rabbits completed treatment, the vulva and vaginal secretions of the rabbits were dynamically observed and scored according to the vulva swelling and the amount of discharge. The scale was as follows: normal (0 point); simple redness and swelling (1 point); redness and swelling with a small amount of white secretions (2 points), significant redness and swelling with a large amount of secretions (3 points), and significant redness and swelling with a large amount of secretions and vaginal rupture and erosion (4 points). The sum of the scores was considered as the degree of inflammation. Then, vaginal lavage fluid was collected, in which a sterile cotton swab was used to wipe the vagina, and then was placed in 1.0 mL of sterilized normal saline. The samples were collected for colony-forming unit (CFUs) assays on the third and seventh day after treatment.

### Histopathological analysis

All the rabbits were euthanized on the seventh day after treatment, and the vaginas were isolated, fixed, and cut into 4-µm thick sections, which were separately stained with Periodic Acid-Schiff’s (PAS) reaction and H&E to observe pathological changes, *Candida* residue, and the glycogen content in the vaginal tissue under an inverted microscope (Nikon, Tokyo, Japan).

### Vaginal apoptosis detection

The vaginal apoptotic cells were detected using a terminal deoxynucleotidyl transferase dUTP nick-end labeling (TUNEL) assay along with DAPI staining to mark the outline of the vaginal tissue. The section was digested with proteinase K and incubated with a TUNEL detection solution according to the manufacturer’s instructions using a one-step TUNEL cell apoptosis detection kit (C1088, Beyotime, Shanghai, China). The apoptotic cells were marked with bright green fluorescence and the distribution of apoptotic cells was observed under a fluorescence microscope.

### ELISA measurement of inflammatory cytokines

The vaginal lavage fluid was collected using a sterile cotton swab to wipe the vagina, which was then placed in 1.0 mL of sterilized PBS (pH 7.2). The fluid was freeze centrifuged at 3000 rpm for 20 min, and the supernatant was separated and stored at − 80 °C. The supernatant was then used for the quantification of the inflammatory cytokines IFN-γ, TNF-α, IL-2, IL-4, IL-6, and IL-17 after completion of different treatment modalities using rabbit IFN-γ, TNF-α, IL-2, IL-4, IL-6, and IL-17 ELISA kits (JingMei BioTech, Jiangsu, China) in accordance with the manufacturer’s instructions.

### Measurement of *C. albicans* intracellular ROS post-treatment

The capability of US-mediated nanoparticles therapy to produce intracellular ROS in *C. albicans* was measured by a DCFH-DA kit (Beyotime Biotechnology, China). Briefly, the *C. albicans* suspensions were incubated with the fluorescent probe DCFH-DA at a 1:1000 dilution (10 μM) at 37 °C for 30 min. Then, *C. albicans* suspensions were treated with AmB or AmB-NPs alone or combined with US irradiation at an intensity of 1.0 W/cm^2^ for 5 min at a 50% duty cycle in pulsed mode. The intracellular ROS of *C. albicans* were quantitatively detected by flow cytometry (FACS Vantage SE, BD Company, USA) within 2 h post-treatment.

### Detection of peroxide and antioxidant enzyme levels in the vagina

The activity of peroxide and antioxidant enzymes in vaginal secretions was analyzed before and after treatment. Rabbit vaginal secretions were collected on day 0 (before treatment) and day 7 (after treatment) of the experimental animal treatment, in which a sterile cotton swab was used to wipe the vagina, and then was placed in 1.0 mL of sterilized PBS, pH 7.2, and freeze centrifuged at 10,000 rpm for 20 min to obtain the supernatant containing enzymes. The expression of peroxide (malonaldehyde, MDA) and antioxidant enzymes (superoxide dismutase, SOD) of the prepared supernatant were tested by a spectrophotometer method using MDA and SOD kits (Nanjing Jiancheng Bioengineering Institute,A005-1, Nanjing, China) according to the manufacturer’s instructions.

### Statistical analysis

Quantitative data in this study were described as the means ± standard deviation (X ± SD) and analyzed using GraphPad Prism version 8 (GraphPad Software; La Jolla, CA, USA). Comparisons between two groups were performed using the *t* test, and comparisons among multiple groups were performed using one-way ANOVA. P < 0.05 was considered to be statistically significant.

## Results

### Characterization of the AmB-loaded nanoparticles and drug release in vitro

**Fig. 2:**
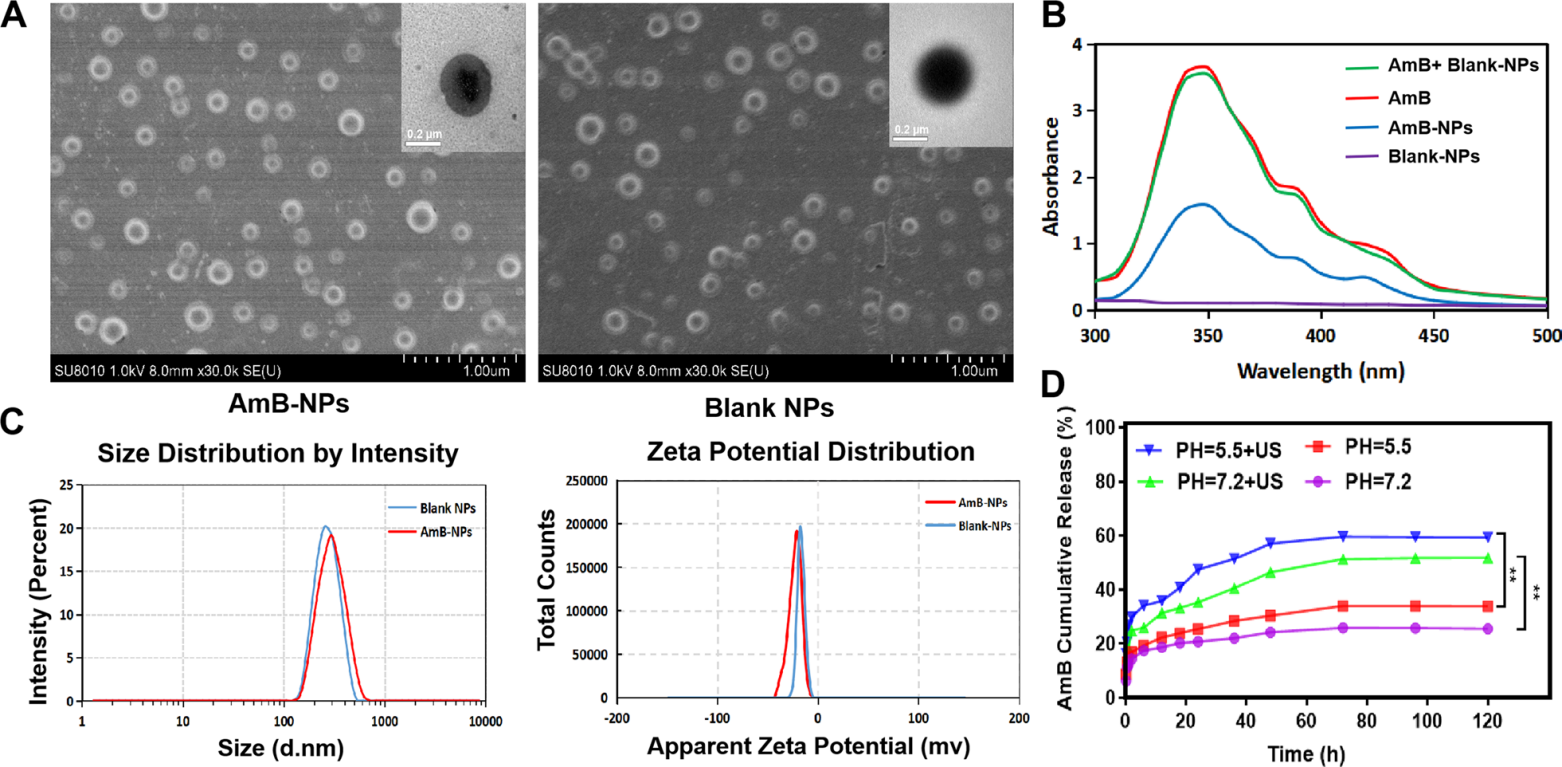
The physicochemical properties, morphology and drug release of the fabricated AmB-NPs. **A** SEM and TEM image of the AmB-NPs and Blank NPs (SEM scale bar = 1 μm, TEM scale bar = 0.2 μm). **B** The absorbtion value of free AmB, AmB-NPs and Blank-NPs at 350 nm. **C** The average size distribution and the zeta potential distribution of AmB-NPs. **D** The percentage of AmB cumulative release from AmB-NPs at different pH with and without US irradiation during 0–120 h incubation. The double asterisk (**) denotes a significant increase (P < 0.01) after ultrasound irradiation

The PEG-PLGA block copolymer with the molecular weight of PEG3500-PLGA15000 was used for preparation of the drug-loaded nanoparticle formulations by the double emulsion method. The physicochemical properties, morphology, and drug release of the fabricated AmB-NP formulations are summarized in Table 1 and Fig. 2. SEM photomicrographs of the prepared AmB-NPs revealed a spherical morphology with uniform particle size. The Blank-NPs were not significantly different from the AmB-NPs in appearance. However, TEM images showed that fine particles could be clearly observed in the center of the AmB-NPs compared to the Blank-NPs, and showed a uniform water-like density shadow (Fig. 2A), which indicated that AmB was successfully encapsulated into the PLGA-PEG shell. In TEM scanning, samples with a higher atomic density block more electrons and result in a darker image [21]. Moreover, the highest absorption peak of the AmB-NPs overlapped with that of the AmB drug, but the Blank-NPs (NPs without the drug) did not show an absorption peak at 350 nm (Fig. 2B), which also indicated that AmB was present in the AmB-NPs system. The average diameter of the AmB-NPs was 252.25 ± 4.59 nm with a PDI of 0.091 ± 0.03 and a mean zeta potential of − 22.0 ± 0.78 mV, both of which indicate a narrow size distribution (Fig. 2C). Moreover, there was no noticeable difference in particle size or zeta potential between the AmB-NPs compared to the Blank-NPs, indicating that the addition of AmB did not affect the distribution of particle size and potential. Finally, the EE% and LC% of AmB in the AmB-NPs were 84 ± 1.5% and 5.1 ± 0.18%, respectively (Table 1).

**Table 1:** The physical characteristics of nanoparticle formulations

Formulations	AD (nm)	ZP (mV)	PDI	LC (%)	EE (%)
Blank NPs	248.63 ± 5.38	− 19.3 ± 1.25	0.069 ± 0.008	−	–
AmB-NPs	252.25 ± 4.59	− 22.0 ± 0.78	0.091 ± 0.03	5.1 ± 0.18	84 ± 1.5
DiI-NPs	261.80 ± 3.62	− 20.8 ± 1.52	0.227 ± 0.039	–	–

*Blank NPs* blank PLGA-PEG nanoparticles, *AmB-NPs* Amphotericin B-loaded PLGA-PEG nanoparticles, *DiI-NPs* DiI fluorescent dye-loaded PLGA-PEG nanoparticles, *AD* average diameter, *ZP* zeta potential, *PDI* polydispersity index, *LC* loading content, *EE* entrapment effciency

The pharmacokinetic duration of a drug in intravaginal drug delivery is inevitably not as long as the duration of intravenous administration, and therefore the ability to facilitate its controlled release is an indispensable property of an efficient AmB delivery system. As shown in Fig. 2D, an immediate and dramatic AmB release from the AmB-NPs stimulated by US exposure within the initial 4 h (about 40%) and a significantly higher AmB accumulative concentration within 120 h was observed after ultrasonic irradiation compared to the non-US exposure group, which demonstrated that the AmB-NP releasing behavior could be mediated by US exposure. In addition, the drug release was higher under acidic conditions (pH 5.5) than under neutral conditions (pH 7.2) regardless of US intervention, which indicated that the constructed nanoparticles were more conducive to intravaginal drug delivery.

### Bio-safety analysis of US-mediated AmB-NPs treatment in vitro and in vivo

**Fig. 3:**
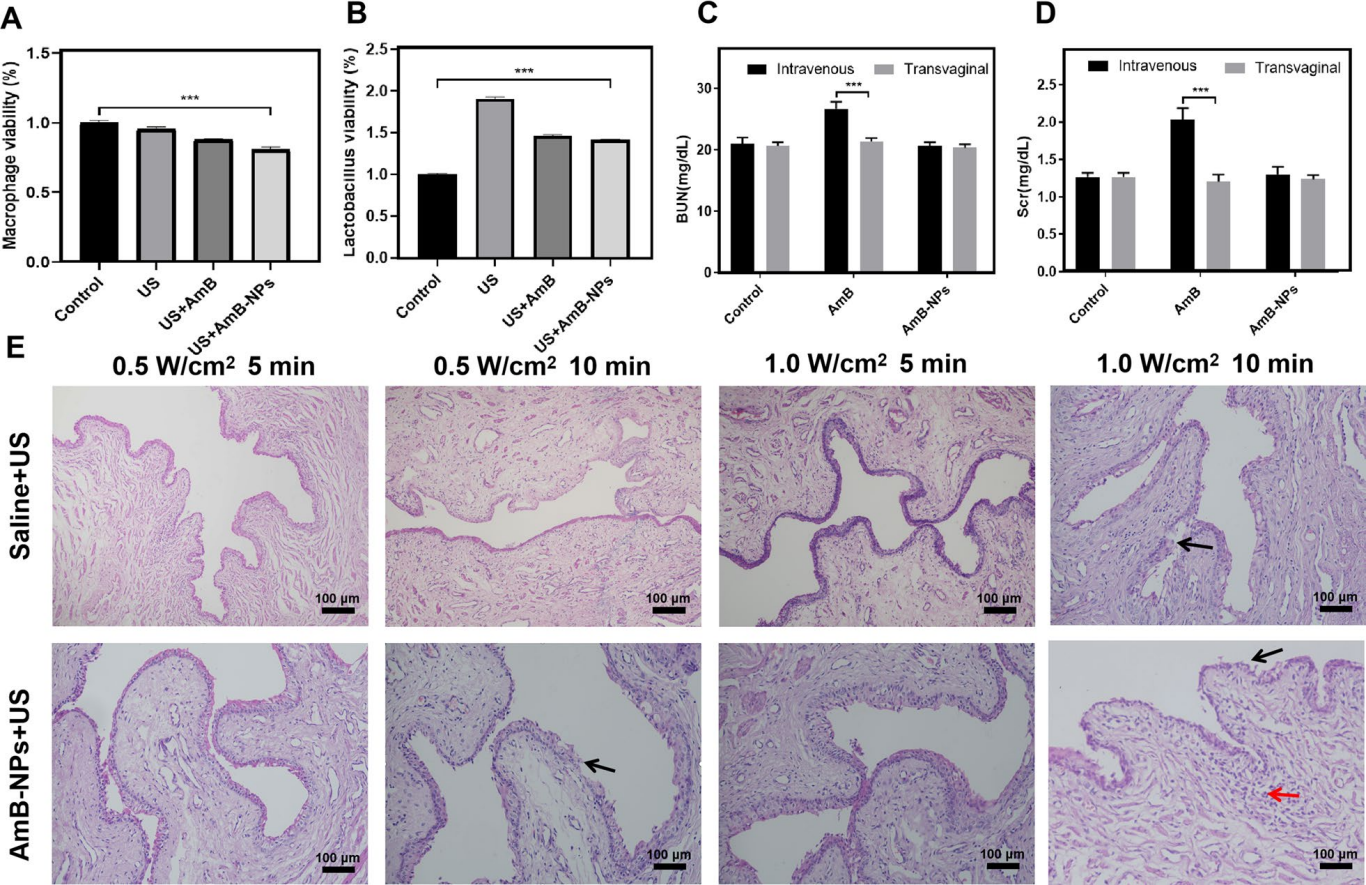
Bio-safety analysis of US-mediated AmB-NPs in vitro and in vivo. The viability changes of macrophages **A** and *L. crispatus* **B** treated with US combined with AmB-NPs at 1.0 W/cm^2^ ultrasonic intensity irradiation for 5 min. The level of plasma urea nitrogen (BUN) **C** and creatinine (SCr) **D** in rabbit following treatment with free AmB and AmB-NPs at a dose of 5 mg/kg/day for three days via intravenously or intravaginally perfused respectively. **E** The pathological structural changes of vagina tissue after US alone or combined with AmB-NPs under different ultrasonic intensity and irradiation time in vivo, black arrows indicate damage to the vaginal mucosa, red arrows show submucosal inflammatory cell aggregation (scale bar = 100 μm). **P < 0.01, and ***P < 0.001

The bio-safety of US-mediated AmB-NPs treatment in vitro and in vivo, from cytotoxicity to vagina pathological structure changes, was evaluated, as depicted in Fig. 3. The activity of macrophages decreased with the increase of ultrasonic intensity and irradiation time, and the cellular activity was higher than 80% under ultrasonic irradiation at an intensity of 0.5 W/cm^2^ and 1.0 W/cm^2^ for 5 min (Additional file 1: Fig. S1A). The activity of cells treated with US combined with AmB-NPs decreased, but the activity remained at 80% (Fig. 3A). In contrast, US irradiation promoted the proliferation of *L. Crispatus* under the low intensities of 0.5 W/cm^2^ and 1.0 W/cm^2^ for 5 min and 10 min (Additional file 1: Fig. S1B). Moreover, the combined treatment of US and the AmB-NPs also promoted the proliferation of *L. crispatus* by 1.4 times compared to the controls, but the amplitude of proliferation was reduced to that of US alone (Fig. 3B). In addition, the AmB-NPs relative to free AmB showed lower cytotoxicity against normal RAW264.7 macrophages and *L. crispatus* at varying equivalent concentrations (1.0, 2.0, 4.0, 8.0, and 16.0 μg/mL) in vitro (Additional file 1: Fig. S2). In an in vivo experiment, the plasma BUN and SCr levels of rabbits treated with AmB-NPs were not significantly increased regardless of intravenous or transvaginal delivery compared to the controls, but both values were significantly increased after intravenous injection of free AmB at the same fivefold clinical dose (P < 0.001) (Fig. 3C, D), indicating that the encapsulated AmB may reduce nephrotoxicity compared to the free AmB drug.

Then, as shown in Fig. 3E, the pathological structural changes of vagina tissue were analyzed after US alone or combined with AmB-NPs under different ultrasonic intensities and irradiation times in vivo. The damage of the tissue mucosa was observed after ultrasonic irradiation alone at 1.0 W/cm^2^ for 10 min, including incomplete mucosa (black arrow) and submucosal inflammatory cell aggregation (red arrow). Similar mucosal damage effects were observed with the combination of the AmB-NPs and US irradiation at 0.5 W/cm^2^ for 10 min. However, no significant damage was observed in the combined group under ultrasonic irradiation at 0.5 W/cm^2^ for 10 min and 1.0 W/cm^2^ for 5 min. Therefore, the ultrasonic irradiation parameters were selected at an intensity of 1.0 W/cm^2^ for 5 min in vitro and in vivo as the safety threshold dose in this study.

### US-mediated AmB-NPs treatment enhanced antifungal activity in vitro

Since the vaginal mucus is the major barrier for the treatment of *C. albicans* VVC infections, the antifungal susceptibility of US-mediated AmB treatment in the presence of mucus was investigate under neutral and acidic conditions, as shown in Table 2. Planktonic *C. albicans* are highly sensitive to AmB, but exhibit resistance when hidden behind the mucus barrier (> 2 μg/mL). Acidic conditions of the vagina impacted the susceptibility to AmB, increasing the MIC twofold, but the antifungal ability of the AmB-NPs was enhanced, presumably because acidic conditions were conducive to drug release from the nanoparticles. Moreover, the antifungal activity of the AmB-NPs under the mucus barrier was comparable to that of AmB, presumably because the penetration ability of the nanoparticles was enhanced after PEG modification. After US treatment, the drug sensitivity was significantly enhanced, and the MIC for AmB was reduced by 2–fourfold. US-mediated AmB-NPs treatment demonstrated a more potent antifungal effect, with a MIC decrease of 4–eightfold, which effectively increased the drug sensitivity even in the presence of the vaginal mucus barrier.

**Table 2:** Antifungal susceptibility of US-mediated AmB and AmB-NPs on *C. albicans* planktonic cells with or without vaginal mucus barrier

	Without vaginal mucus				With vaginal mucus			
	US (−)		US ( +)		US (−)		US ( +)	
	MIC	MFC	MIC	MFC (mg/l)	MIC	MFC	MIC	MFC
pH 7.2								
AmB	0.5	1	0.25	0.5	4	8	1	2
AmB-NPs	0.5	1	0.125	0.25	2	4	0.5	1
pH 5.5								
AmB	1	2	0.5	1	8	16	2	4
AmB-NPs	0.5	1	0.125	0.25	2	4	0.5	1

*US* ultrasound, *MIC* minimal inhibitory concentration, *MFC* minimum fungicidal concentration, *AmB* Amphotericin B, *AmB-NPs* Amphotericin B-loaded PLGA-PEG nanoparticles

### Permeability of US-mediated nanoparticles treatment on mucus and vaginal tissue

**Fig. 4:**
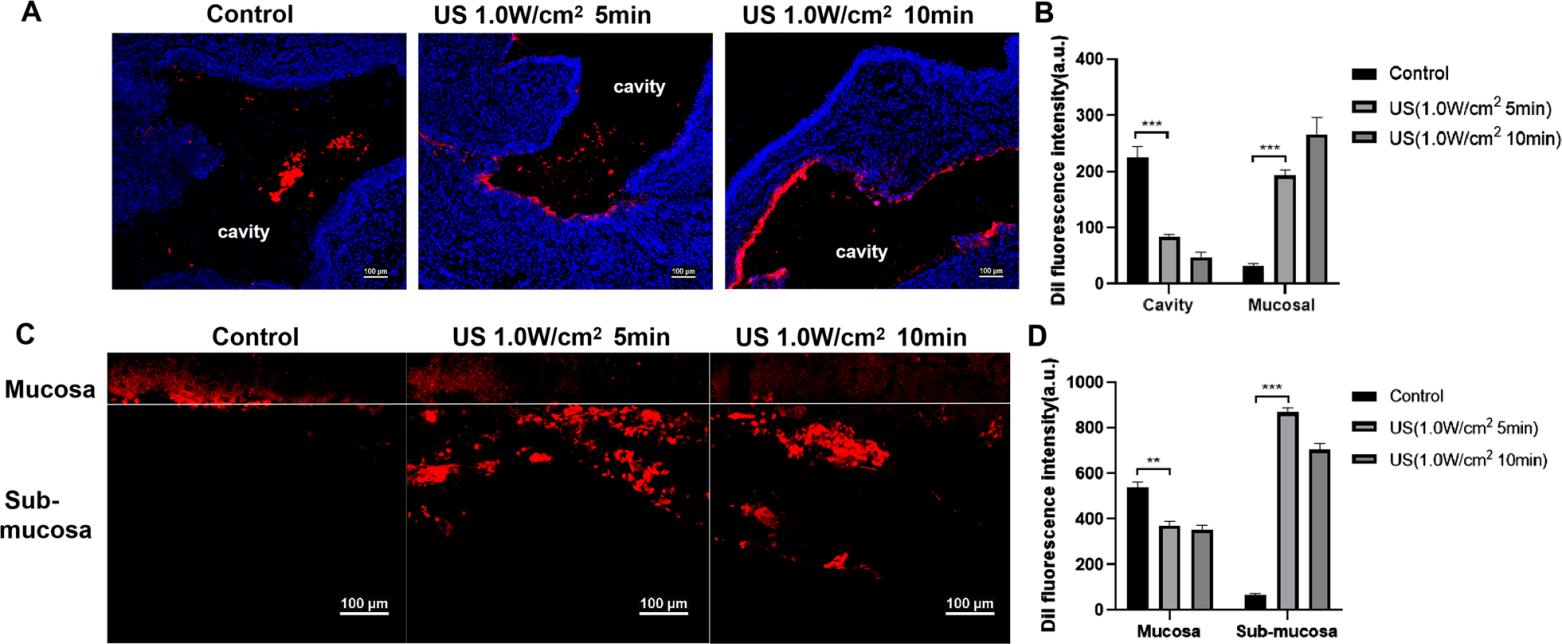
Permeability analysis of US-mediated nanoparticles on mucus and vaginal tissue. **A** The distribution of DiI red fluorescent nanoparticles in the vaginal cavity with or without US irradiation observed by CLSM (scale bar = 100 μm). **B** The quantitative calculation of the fluorescence intensity in the vaginal cavity and mucosal. **C** Observation of DiI nanoparticles penetration in vaginal tissue in vivo (scale bar = 100 μm), the nanoparticles were mainly distributed in the submucosal area after ultrasound irradiation but that were concentrated in the mucosal layer in the control group. **D** Quantitative analysis of permeability through the average area of red fluorescence intensity (5 randomly-selected view fields) in the mucosal layer and submucosal area of vagina tissue. ***P < 0.001

To explore the impact of US on the distribution of nanoparticles in vivo, the infiltration and distribution of nanoparticles into mucus and vaginal tissue was observed via LSCM, as shown in Fig. 4. A large number of nanoparticles aggregated in the cavity of the mucus layers and did not penetrate into the vaginal rugae in the controls, while the nanoparticles were mainly distributed in the vaginal mucosal surfaces and formed a continuous particle layer that coated the entire vaginal epithelium after US irradiation (Fig. 4A). Further quantitative calculation of the fluorescence intensity in the vaginal cavity and mucosa showed that the average area red fluorescence intensity in the vaginal mucosal area was significantly higher than that in the vaginal cavity after US irradiation (Fig. 4B). Furthermore, we observed the presence of nanoparticles in the vaginal tissue. Clearly, the nanoparticles were mainly distributed in the submucosal area after US irradiation but were concentrated in the mucosal layer (distribution depth less than 60 μm) in the control group, as shown by a longitudinal profile of the vaginal tissue after LSCM scanning (Fig. 4C), and the quantitative analysis showed that the fluorescence intensity group in the submucosal area of the US group was significantly higher than that of the control group (Fig. 4D). These results demonstrated that US-mediated nanoparticles significantly increased the permeability in the mucus and promoted the distribution of drugs to the submucosal area.

### Synergistic therapeutic effect of US-mediated AmB-NPs treatment in vivo

**Fig. 5:**
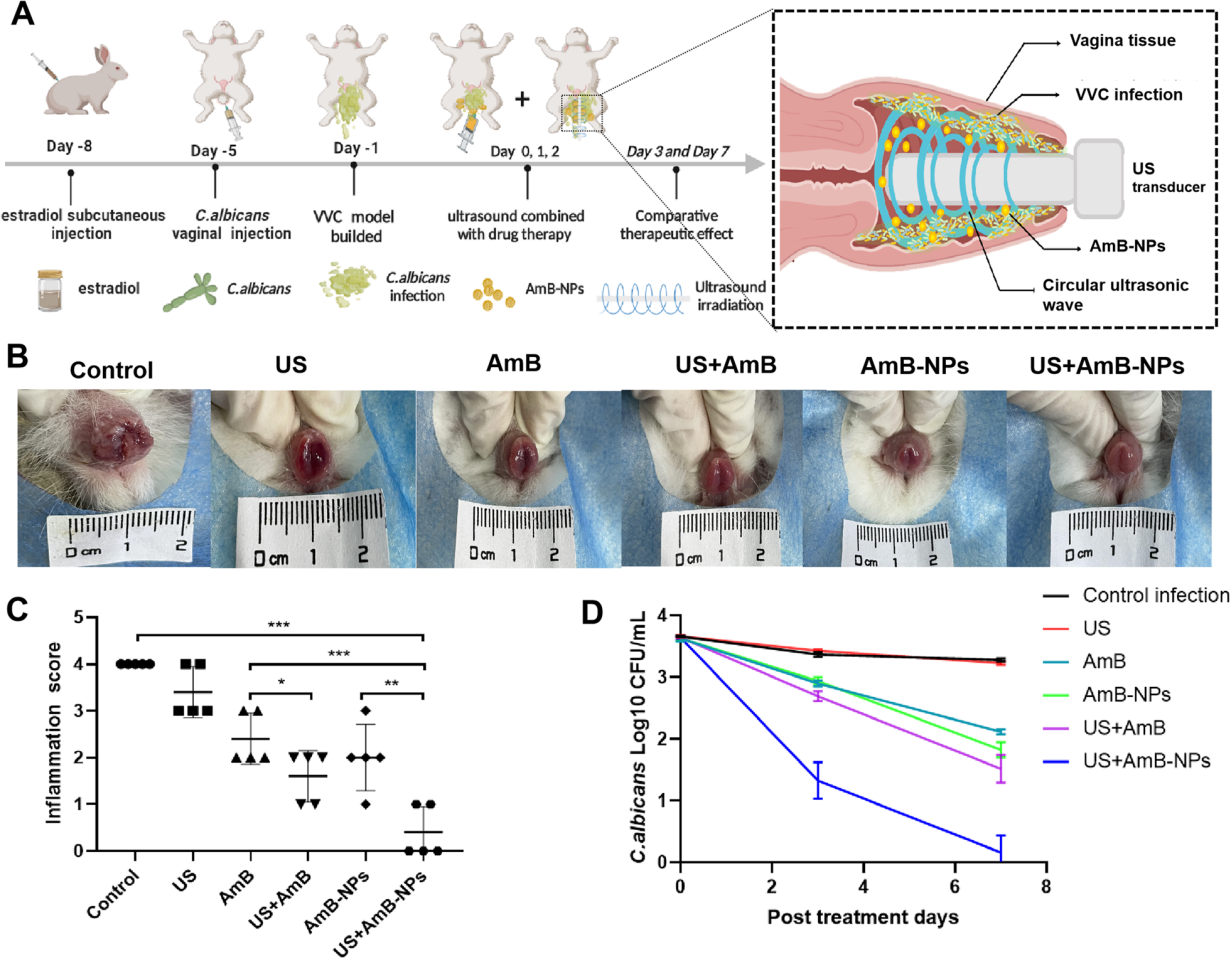
Synergistic therapeutic effect of US-mediated AmB-NPs for treatment of VVC in rabbit model. **A** The timeline of the experimental animal treatment and the schematic illustration of US-mediated nanoparticles therapy in vagina. **B** Macroscopic observation of vaginal vulva redness, swelling and vaginal secretions on seventh day period after different treatment. **C** The inflammation score was calculated in different group base on the vulva hyperemia, redness and swelling, mucosal ulceration and white secretion. **D** Quantitative analysis of fungal colony counts (Log10 CFU/mL) of the vaginal lavage fluid in animals infected with *C. albicans* on the zero, third, and seventh day post-treatmen. Data represent mean ± standard deviation of initial vaginal fungal burden (n = 5 per group). ***P < 0.001. **P < 0.01

To investigate the therapeutic effect of US-mediated AmB-NPs treatment in vivo, treatments combining ultrasound and AmB-NPs were conducted on the experimental VVC model of rabbits, as shown in Fig. 5A. The rabbit VVC model was successful established on the fifth day after continuous injection of a *C. albicans* solution into the vagina with inflammatory symptoms and fungi colonization (Additional file 1: Fig. S3). On the seventh day after different treatments, the vulva was hyperemic, with redness and swelling, mucosal ulcerations, and increased white secretions in the control group, corresponding to a four inflammation score. However, after treatment with ultrasound combined with AmB-NPs, the vulva inflammatory symptoms were clearly relieved, and no redness, swelling, or white secretions were observed, corresponding to a 0–1 inflammation score (Fig. 5B). Similarly, the quantitative analysis of the inflammation score in the US + AmB-NPs group was significantly decreased (Fig. 5C). Then, the fungus colonies in the infected vagina were quantified and presented as log_10_ CFU/mL on the zero, third, and seventh day post-treatment, as shown in Fig. 5D and Table 3. After joint treatment of ultrasound with AmB-NPs, only one rabbit remained single digit CFU-infected (N 1/5, 20%) after seven days of local treatment, and the average fungal burden (0.159 ± 0.275) was significantly lower than that of the control group (3.642 ± 0.041) (P < 0.01), which indicated that US-mediated AmB-NPs treatment effectively removed *C. albicans* colonized in the vagina. While US treatment alone showed no significant antifungal effect compared to the control group (P > 0.05).

**Table 3:** Vaginal fungal burden in different treatment group (Log CFU mL-1 ± SD and number of infected rabbits /original infected rabbits, n = 5 per group)

Group	0 day (%)	3 day (%)	7 day (%)
Control	3.661 ± 0.010 (N 5/5, 100)	3.367 ± 0.039 (N 5/5, 100)	3.278 ± 0.220 (N 5/5, 100)
US	3.630 ± 0.036 (N 5/5, 100)	3.428 ± 0.018 (N 5/5, 100)	3.227 ± 0.029 (N 5/5, 100)
AmB	3.628 ± 0.047 (N 5/5, 100)	3.897 ± 0.048 (N 5/5, 100)	2.114 ± 0.530* (N 4/5, 100)
US + AmB	3.643 ± 0.010 (N 5/5, 100)	2.690 ± 0.081 (N 4/5, 80)	1.513 ± 0.223* (N 2/5, 40)
AmB-NPs	3.632 ± 0.045 (N 5/5, 100)	2.939 ± 0.063 (N 4/5, 80)	1.820 ± 0.124* (N 3/5, 60)
US + AmB-NPs	3.642 ± 0.041 (N 5/5, 100)	1.325 ± 0.297* (N 2/5, 40)	0.159 ± 0.275** (N 1/5, 20)

*P < 0.05, **P < 0.01

### Histological analysis

**Fig. 6:**
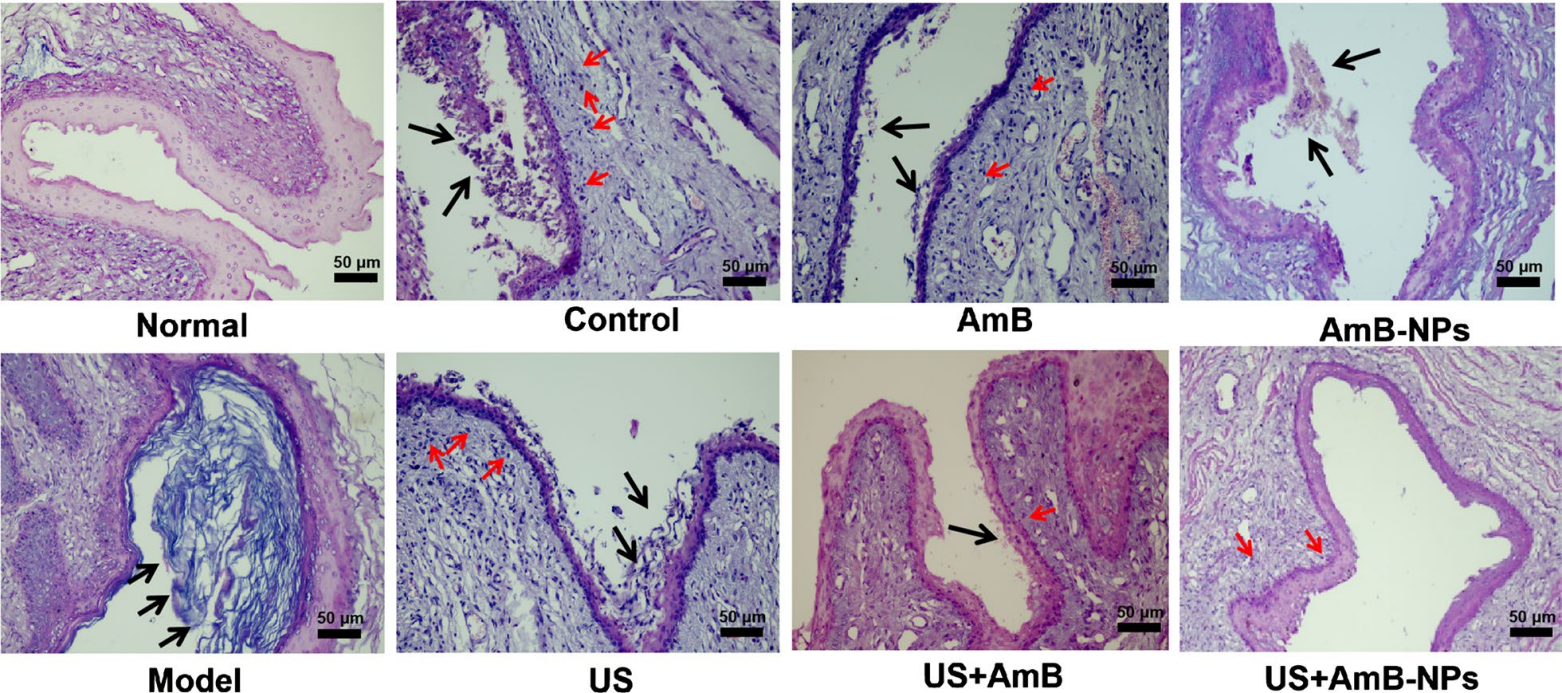
Histopathological and inflammatory changes of rabbit vaginas were observed by H&E staining (scale bar = 50 μm). The established VVC model vagina shown a biofilm-like structure formed by dense hyphae and *C. albicans* cells adhered to the mucosa (black arrows). On the seventh day period after different treatment, the control group also shown numerous colonies of hyphae in the vaginal cavity and further aggravated inflammatory symptoms with incomplete mucosa structure, inflammatory cells (red arrows) gathered and distributed under the mucosa. The AmB related treated group improved inflammatory infiltration. After US + AmB-NPs treatment, no obvious colonies and hyphae were residual in the cavity and the submucosal inflammatory cells were significantly reduced

Histological analysis using H&E staining showed a clear difference between vaginal tissues of healthy and *C. albicans*-infected animals subjected to different treatments, as indicated in Fig. 6 and Additional file 1: Fig. S4. Compared with the normal group, the successfully established VVC model vagina showed a biofilm-like structure formed by dense hyphae, with *C. albicans* cells adhered to the mucosa and partially invading the submucosa. The vaginal epithelium showed intense inflammatory infiltrates that comprised polymorphonuclear cells. On the seventh day after treatment, the inflammatory symptoms in the control group (treated with sterile saline solution) were further aggravated with an incomplete mucosa structure, the number of epithelial cells had decreased, and a large number of inflammatory cells gathered and distributed under the mucosa. US treatment alone also showed histopathological characteristics typical of infection with hyphae around the vaginal epithelium. After treatment with AmB or AmB-NPs alone, obvious colony hyphae residue in the vaginal cavity and submucosal inflammatory cells were still observed. The US + AmB group showed a reduced presence of hyphae and inflammatory cell infiltration. However, after US jointly with AmB-NPs treatment, no obvious residual colonies or hyphae were present in the cavity, the vaginal wall structure was complete with clear layering, and the submucosal inflammatory cells were significantly reduced, which was not significantly different from the normal vaginal structure.

### Effect on glycogen content in the vagina

**Fig. 7:**
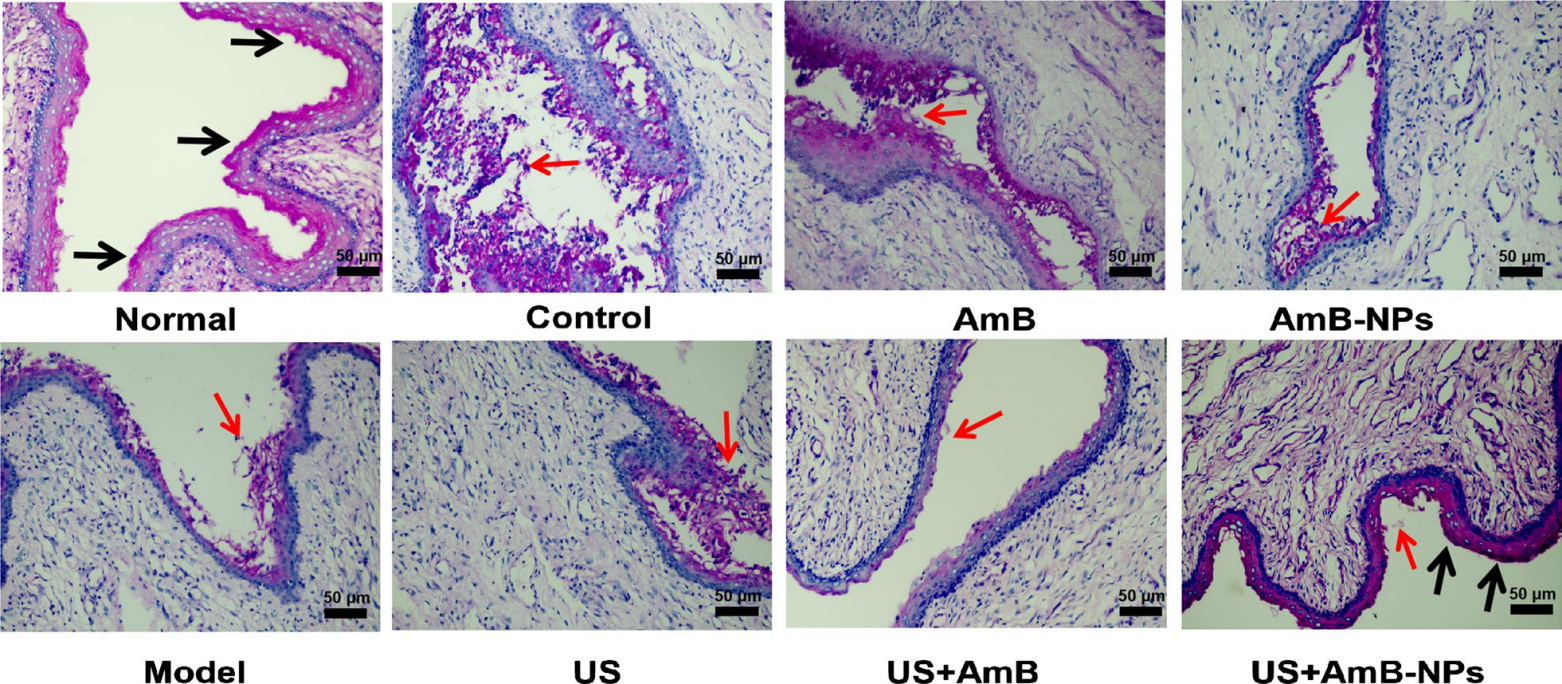
PAS glycogen staining observed the glycogen content in the vaginal epithelial after treatment in each group (scale bar = 50 μm). (black arrows indicate large amount of glycogen expression and evenly covered on the mucosal surface; red arrows indicate *C. albicans* cells and hyphae the vagina)

Normally, glycogen expressed by the lower genital tract epithelial cells is believed to support *Lactobacillus* growth and play an important role in maintaining the normal vaginal environment in vivo. PAS glycogen staining was used to analyze the glycogen content in the vaginal epithelial after treatment, as shown in Fig. 7 and Additional file 1: Fig. S5. Compared to the normal vaginal epithelium with a large amount of glycogen expression evenly covering the mucosal surface, a significant decrease of glycogen content was visible in the constructed VVC infection model and the control group (treated with sterile saline solution). Glycogen expression was not significantly improved by US irradiation alone, and glycogen levels were slightly and unevenly increased after treatment with AmB or AmB-NPs alone. However, after combined treatment of US with AmB-NPs, glycogen levels were clearly increased and dense and uniformly distributed on the surface of the vagina mucosa, and showed no significant difference in glycogen content and distribution compared with the normal vaginal epithelium.

### Vaginal apoptotic cell observation

**Fig. 8:**
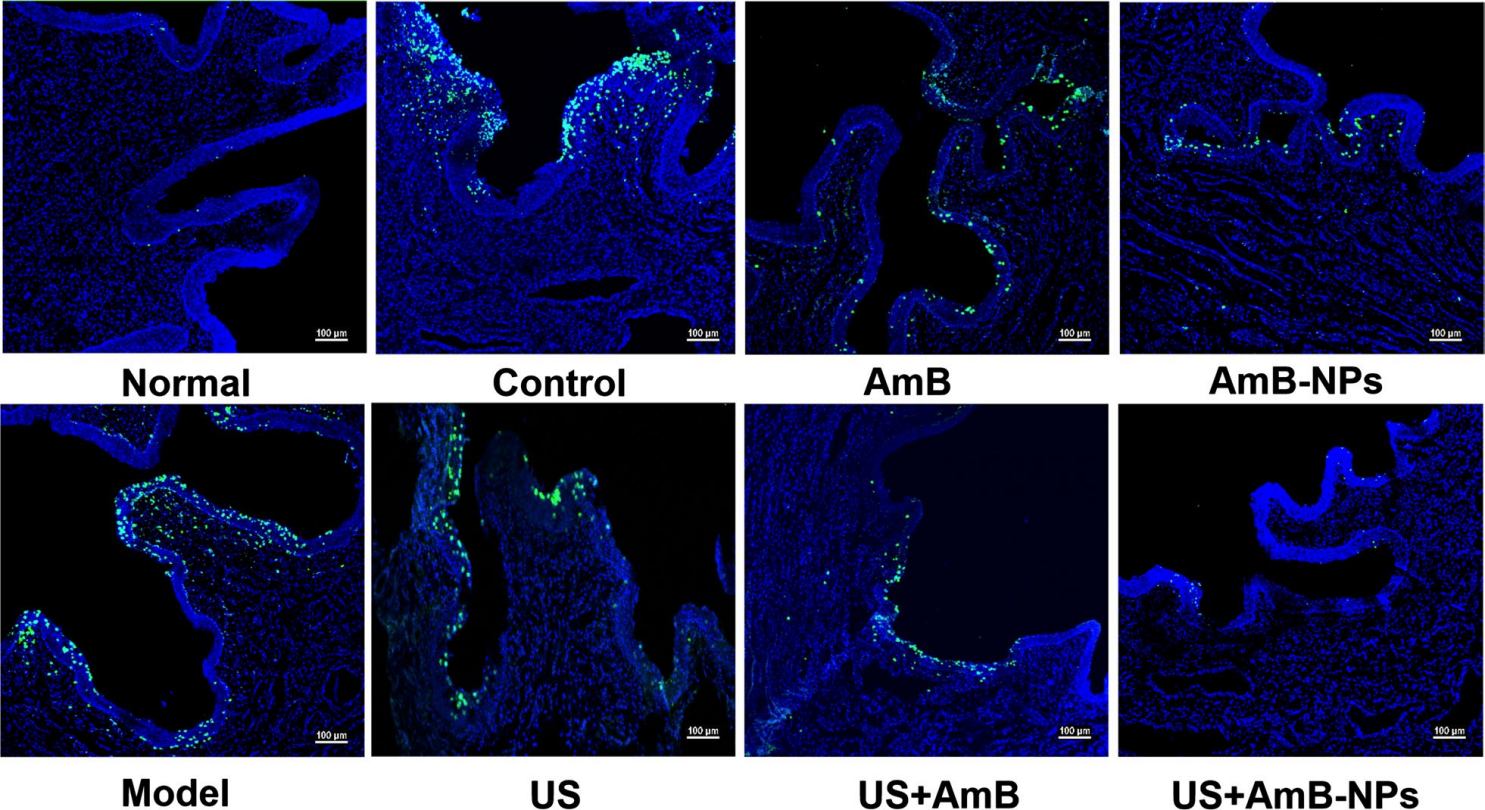
The detection of vaginal apoptotic cells after treatment in each group using TUNEL staining. The apoptotic cells were marked with bright green fluorescence and the outline of vaginal tissue was shown by DAPI staining (scale bar = 100 μm)

*Candida* colonization in the vagina induces apoptosis of vaginal cells, which is consistent with the progression of inflammation. Vaginal apoptotic cells were detected using TUNEL staining after treatment in each group, as shown in Fig. 8. A large number of apoptotic cells showed as green fluorescence in the vaginal tissues of the VVC rabbits (model group), and the positive expression of apoptotic cells further increased on the seventh day after infection (control group). After treatment with drugs and US, the expression of apoptotic cells in the vagina decreased unevenly. Particularly with US combined with AmB-NPs treatment, only a few apoptotic cells were found, indicating that the infection was significantly improved.

### Quantification of cytokines in vaginal secretions

**Fig. 9:**
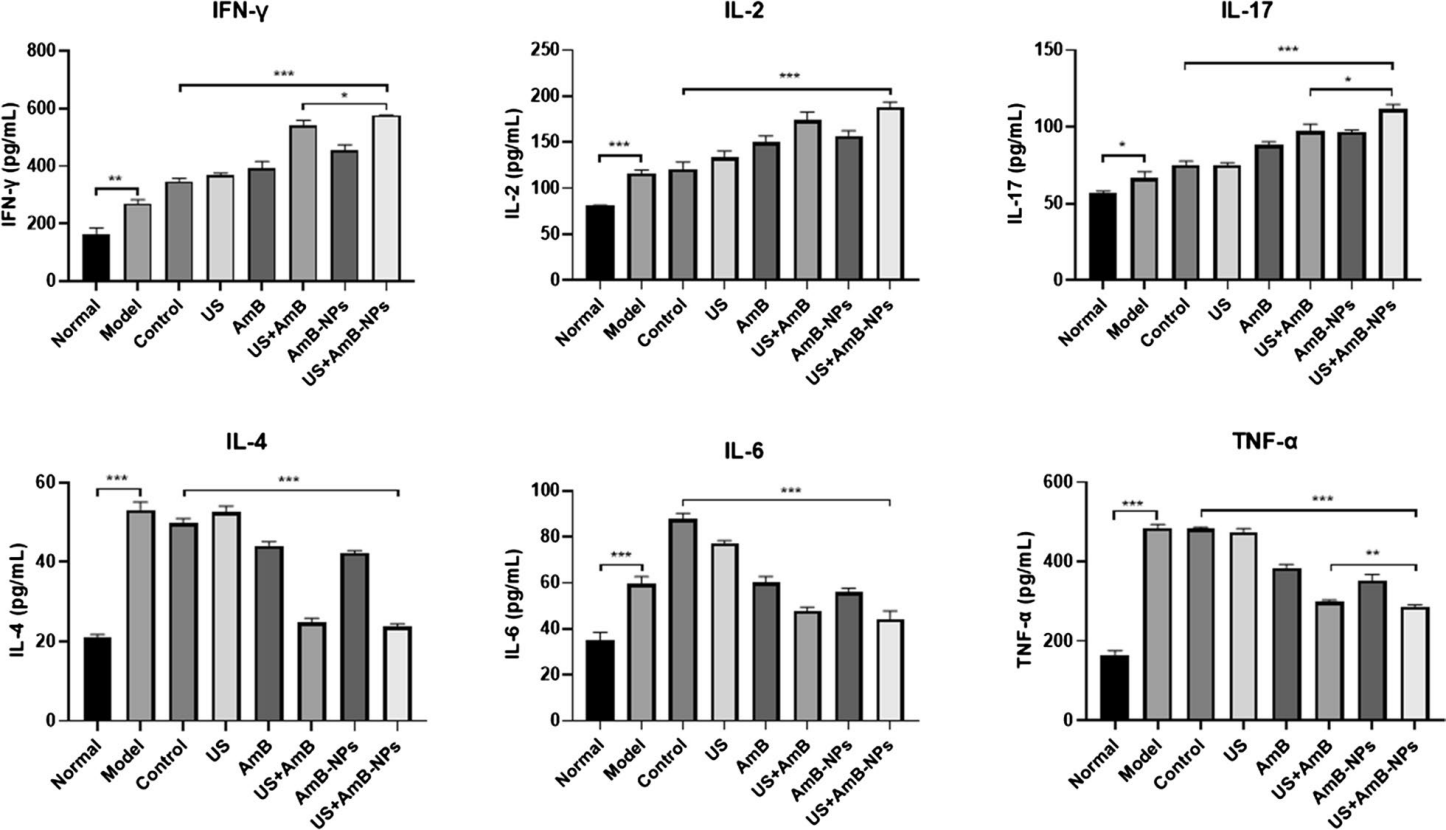
The expression of IFN-γ, IL-2, IL-17, IL-4, IL-6, and TNF-α were determined in the vaginal secretions after completion of different treatment modalities. **P < 0.01. ***P < 0.001

To analyze the inflammatory response triggered by vaginal infection with *C. albicans* and to compare between the treatment groups, the expression of IFN-γ, TNF-α, IL-2, IL-4, IL-6, and IL-17 were determined in the vaginal secretions after completion of different treatment modalities, as shown in Fig. 9. Compared to the healthy rabbits, the levels of the detected cytokines were all significantly increased in the model group and the control group (P < 0.01). On the seventh day after treatment, the mucosal immune factors underwent significant changes compared with pre-medication levels. Specifically, treatment with US alone did not show an effect on the cytokine levels compared to the controls (P > 0.05), but treatments with AmB, AmB-NPs, US + AmB, and US + AmB-NPs were able to reduce IL-4, IL-6 and TNF-α levels, and the expression of these three cytokines was the lowest in the group receiving US jointly with AmB-NPs treatment, which was close to the expression of healthy rabbits. In contrast, the levels of IFN-γ, IL-2 and IL-17 in the AmB, AmB-NPs, US + AmB, and US + AmB-NPs groups were further increased after treatment, particularly in the US + AmB-NPs group, which was significantly higher than in the controls (P < 0.001).

### US-mediated AmB-NPs treatment promoted intracellular ROS generation of *C. albicans*

**Fig.10:**
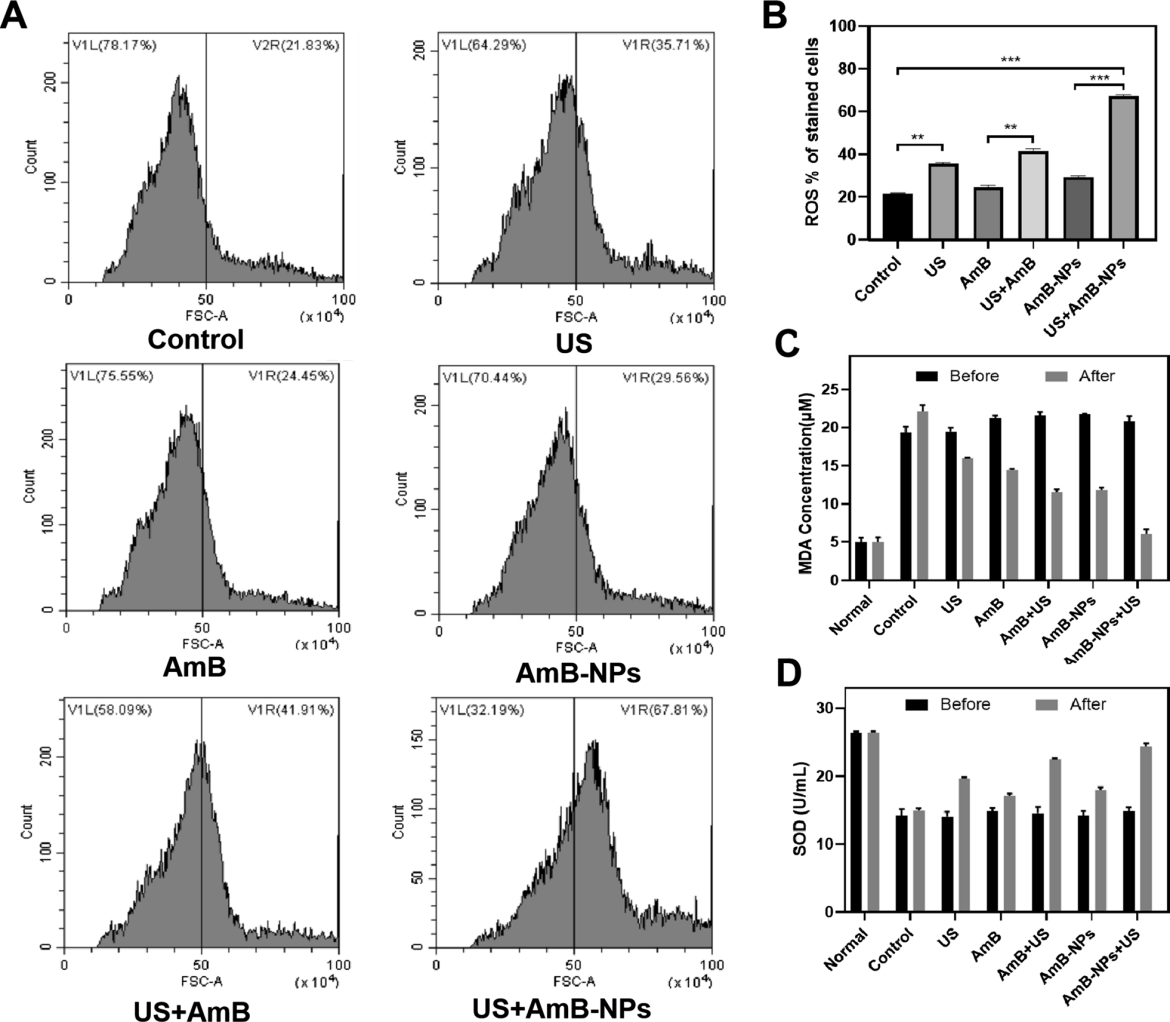
The intracellular ROS of *C. albicans* treated with different modalities were quantitatively detected by flow cytometry within 2 h post treatment (**A**). The percent ROS generation of *C. albicans* stained cells in different group (**B**). The changes of the content of MDA (**C**) and the activity of SOD (**D**) in vaginal secretions on day 0 (before treatment) and day 7 (after treatment). n = 5 per group, **P < 0.01, and ***P < 0.001

The accumulation of excessive intracellular ROS in *C. albicans* can lead to damage of mitochondria and other organelles, further inducing the apoptosis of the thalli. Within 2 h after treatment, intracellular ROS levels were significantly increased following treatment with US-mediated free AmB or AmB-NPs compared with AmB or AmB-NPs alone groups (P < 0.05) (Fig. 10A), which indicated that AmB generated ROS triggered by US. Moreover, the percentage of ROS in *C. albicans* stained cells in the US + AmB-NPs group was higher than in the US + AmB group (Fig. 10B), which indicated that nanoparticles induced a stronger sonochemical reaction to enhance the efficiency of ROS generation upon an US trigger.

### Analysis of oxidative stress status in vaginal secretions

To analyze the oxidative status of the vaginal secretions, the levels of MDA and the activity of SOD were measured before and after treatment. As shown in Fig. 10C, The MDA level in the control group (before treatment) was 19.32 ± 2.14 μM, which is almost four times the normal level in rabbits. Since MDA is a major product of lipid peroxide degradation, the significantly higher concentration of MDA suggested that a serious oxidative stress environment developed in the VVC-infected vagina. However, the rabbit vaginal MDA level was effectively decreased to a level close to a normal level after US jointly with AmB-NPs treatment. In contrast, the SOD activity of vaginal secretions after *C. albicans* infection (14.24 ± 0.82 U/mL) was significantly lower than that of the normal vagina (26.44 ± 3.88 U/mL) (P < 0.01), indicating a decrease in antioxidant activity in the VVC infection. However, after US combined with AmB-NPs treatment, the SOD activity was 24.35 ± 2.14 U/mL, similar to the control level (Fig. 10D). The results demonstrated that the synergistic therapy scheme of US-mediated AmB-NPs treatment could be beneficial for the recovery of the oxidative stress imbalance in the VVC-infected vagina.

## Discussion

Vulvovaginal candidiasis is an infection caused by *Candida* species that affects millions of women every year, which deserves special attention because it is highly recurrent and difficult to completely treat [26]. Intravaginal local treatment is the first recommended treatment for vaginitis, but most of conventional drugs are easily trapped by vaginal mucus, blocking deeper penetration in vagina tissues, and resulting in recurrence susceptibility [27]. In this study, we developed polymeric nanoparticles based on PLGA-PEG incorporating the antifungal AmB and combined low intensity ultrasound-mediated AmB-loaded nanoparticle intravaginal drug delivery to achieve productive, synergistic antifungal activity in a rabbit model of VVC and effectively improve the local immune status.

The development of nanoparticle-mediated drug delivery systems may provide the possibility to reduce drug toxicity and local application of AmB in the vagina [28]. In this study, the prepared AmB-loaded nanoparticles showed lower cytotoxicity against an important immune cell, macrophages, and against vaginal-dominant bacteria *Lactobacillus* in vitro compared to free AmB at the same drug concentration (Additional file 1: Fig. S2). The renal impairment (indicated by plasma BUN and SCr levels) was lower in the AmB-NPs group at a fivefold clinical dose intravenous administration (Fig. 3C, D), which may be because the drug was released slowly and continuously from the nanoparticle formulation to avoid acute kidney in vivo [29]. Furthermore, no obvious pathological structural damages were observed in the mucosa and submucosa of normal vagina tissue in the combined treatment group at an intensity of 1.0 W/cm^2^ for 5 min (Fig. 3E), which verified the biosafety of US-mediated AmB-NPs intravaginal drug delivery in the vagina. The therapy scheme designed for vaginal use must not negatively impact the vaginal environment and survival of *Lactobacillus*, as it is critical that the treatment schemes do not increase the susceptibility to VVC [30].

It is known that vaginal mucus is a protective barrier limiting drug penetration and absorption, and the effect of local applications of nanoparticle formulations in the vagina are closely related to its physical characteristics [9]. The diameter of the AmB-NPs is around 250 nm, which is in accordance with the characteristics desired for mucoadhesive formulations, as particle diameters between 50 and 300 nm present better characteristics for binding to mucosal tissues [31]. Vaginal mucin is negatively charged due to the high density of glycans, which allow mucins to capture any particulates that are overall cationic or possess substantial cationic domains [32]. In the case of combined electrostatic and steric stabilization, a minimum ZP of ± 20 mV is desirable. The formulated AmB-NPs have a negative charge of − 22.0 ± 0.78 mV, which effectively avoids electrostatic adsorption and immobilization of the nanoparticles in the mucus [33]. The low molecular weight PEG-coating on the surface of the nanoparticles further effectively blocks mucins from interacting with the underlying particle core while also minimizing mucoadhesion based on interpenetrating network effects [13]. In addition to enhancing particle diffusion to overcome the mucus barriers by using ideal physical properties of the nanoparticles, we then combined low-intensity ultrasound with the medicated nanoparticles to generated an intense shear force to enhance drug penetration into the mucus. The result of LSCM scanning observations showed that the drug was mainly distributed in the vaginal mucosal surfaces and formed a continuous particle layer that coated the entire vaginal epithelium after ultrasonic irradiation, but the nanoparticles were aggregated in the cavity mucus layers in controls (Fig. 4A). In addition, ultrasonic irradiation also promoted the distribution of drugs to the submucosal area (Fig. 4C), which provided the possibility for the thorough removal of submucosal pathogenic fungus after ultrasonic irradiation. The mechanism of ultrasound-enhanced membrane permeability mechanism may primarily associated with the cavitation effect with the accompanying extreme physical phenomena, such as shock waves, high shear forces, and microjets, which contribute to break the drug penetration barrier and trigger drug release [34].

Mucus barrier design is a prerequisite for detecting antimicrobial susceptibility of drugs to *C. albicans* under the mucus layer. We compared the antifungal susceptibility of US with AmB formulations in the presence of mucus in vitro. As expected, the mucus barrier increased the MIC of AmB eightfold, significantly enhancing drug resistance. In contrast, US irradiation significantly reduced the barrier resistance and reduced the MIC value, especially in US-mediated AmB-NPs treatment, in which the MIC decreased by 4–eightfold (Table 2), and exhibited significant synergistic antifungal effects even in the presence of the vaginal mucus barrier. In fact, the biological effects of the US-mediated nanoparticles treatment may be different from that of US treatment alone. Nanoparticles as exogenously cavitating nuclei greatly reduced the threshold of localized cavitation and enhanced the cavitation effect [14]. US acoustic cavitation is an extremely violent physical process and produces shock waves, shear forces, and microjets which break the drug penetration barrier and cause inactivation of microorganisms [35, 36]. US also activated sonosensitizer molecules to generate toxic (ROS) compounds that had significant antimicrobial activity against planktonic and biofilm forms [37]. In our study, higher levels of *C. albicans* intracellular ROS were detected within 2 h after US combined with AmB treatment formulations (Fig. 10), suggesting that US activated AmB to increase the ROS content and thus enhanced the antifungal effect. The peak absorbance of AmB (λ max at 350, 381, and 405 nm) correlated well with the maximum emission of sonoluminescence (250–600 nm), which is an important property of compounds that can be activated by US to produce sonodynamic effects [38]. Therefore, the possible mechanisms of US-mediated AmB-NPs promoting intracellular ROS generation of *C. albicans* could be summarized into acoustic cavitation-induced drug penetration enhancement and ROS production related to sonoluminescence [39, 40].

Then, the antifungal activity of US-mediated nanoparticles treatment was investigated with a rabbit model of VVC by multiple methods. The rabbit serves as the gold standard model for FDA-mandated preclinical assessment of vaginal irritation; therefore, such studies often use rabbit vaginal tissue models [37]. After joint treatment of US with AmB-NPs, the average fungal burden of the vaginal lavage was significantly lower than that of other treatments, and only one rabbit remained single digit CFU-infected (N 1/5, 20%) at the seventh day of antifungal therapy (Table 3), which indicated US-mediated AmB-NPs treatment effectively removed *C. albicans* colonized in the vagina. Histological analysis revealed that the structural integrity of the vaginal mucosa was restored, the submucosal inflammatory cells were significantly reduced, and glycogen levels were clearly increased in the vaginal wall. These results indicate that US-mediated AmB-NPs treatment of VVC not only contributed to the removal of pathogenic bacteria, but also promoted the recovery of the structure and function of the vaginal mucosa.

To further analyze the effect of US-mediated nanoparticles on local immunity changes in the vaginas of VVC rabbits, cytokine production associated with Th1 (IFN-γ, IL-2), Th2 (IL-4, TNF-α) and Th17 (IL-17) responses were quantified in the vaginal secretions after completion of different treatment modalities. Generally, Th1 cytokines induce protective mucosal immunity responses, and Th17 is also essential for mucosal defense against *C. albicans* by induction of antimicrobial peptides, while Th2 responses correlate with disease exacerbation and pathological damage [41, 42]. In our present study, after *C. albicans* infection, the expression of IFN-γ, IL-2, IL-4, TNF-α, and IL-17 cytokines were all significantly increased in the model group and the control group compared to the healthy rabbits, which may be explained by the fact that a relatively high level of cytokines is a prerequisite for activating a protective mucosal response or maintaining homeostasis [43]. On the seventh day after treatment, expression levels of the cytokines IFN-γ, IL-2, and IL-17 were significantly increased after joint treatment of US with AmB-NPs, but expression levels of IL-4 and TNF-α were significantly reduced compared with the control group (Fig. 9). The results demonstrated that US-mediated nanoparticles treatment of VVC might provide a protective immune response by promoting the shift of the vaginal local immune status toward the Th1- and Th17-type, thereby helping to eliminate local *C. albicans* and inhibiting the occurrence and development of inflammation.

In addition, increased peroxides and decreased antioxidant enzyme activity are the manifestations of an oxidative stress imbalance in vaginitis, which limits the recovery of vaginal inflammation to a certain extent [44, 45]. In this study, high concentrations of MDA and reduced SOD activity were observed in vaginal discharge after *C. albicans* infection, indicating that oxidative stress was formed in the VVC vaginal microenvironment. Furthermore, we analyzed the oxidative status of the vaginal secretions before and after treatment in different groups, and the results demonstrated that the synergistic therapy scheme of low intensity ultrasound combined with AmB-NPs reduced MDA levels and increased the activity of SOD, which was beneficial for the recovery of the oxidative stress imbalance in the VVC-infected vagina to a normal state. However, the specific causes and pathways affecting oxidative stress still need to be further studied in the future.

## Conclusion

In this study, AmB-loaded PEGylated PLGA nanoparticles were developed and characterized, which showed lower host toxicity with an ultrasound-sensitive slow drug release. Then, intravaginal low-intensity ultrasound was combined to further improve the permeability in the vaginal mucus barrier and exhibited significant synergistic antifungal activity in a rabbit model based on VVC assays. The specific manifestation included: (1) efficiently removed *C. albicans* from vaginal colonization and relieved inflammatory infiltration; (2) repaired the pathological damage to the vaginal mucosa, reduced cell apoptosis, and increased glycogen levels in the vagina; and (3) provided a protective immunity response by promoting a shift of the vaginal local immune status toward the Th1- and Th17-type, and was beneficial for the recovery of the oxidative stress imbalance in the VVC-infected vagina to a normal state. This synergistic therapy scheme based on ultrasound-mediated drug-loaded nanoparticle delivery may provide a new non-invasive, safe, and effective therapy for acute or recurrent fungal vulvovaginitis.

## Supplementary Information

**Additional file 1: Fig. S1.** The viability effect of ultrasound on macrophages **A** and *L. crispatus* **B** treated at intensity of 0.5 W/cm^2^, 1.0 W/cm^2^ and irradiation time of 5 min, 10 min, 15 min in 50% duty cycle of pulse mode. **Fig. S2.** The comparison of macrophages viability **A** and *L. crispatus* viability **B** treated with free AmB and AmB-NPs at different concentrations of AmB from 0 to 16 μg/mL. **Fig. S3** Observation of constructed vulvovaginal candidiasis model in rabbits after 3 days and 5 days of continuous injection of *C. albicans* solution into the vagina by Macroscopical, Gram staining (bar = 10 μm), HE staining (bar = 100 μm) and *C. albicans* culture of vaginal lavage fluid on SDA. **Fig. S4.** H&E stained images of rabbit vaginas conducted by different treatments (scale bar = 20 μm, 40 × magnification). **Fig. S5.** PAS stained images of rabbit vaginas conducted by different treatments (scale bar = 20 μm, 40 × magnification).

## Data Availability

The data, analytical methods, and study materials for the purposes of reproducing the results or replicating procedures can be made available on request to the corresponding author who manages the information.
