# Supplementary material for: Low intensity ultrasound-mediated drug-loaded nanoparticles intravaginal drug delivery: an effective synergistic therapy scheme for treatment of vulvovaginal candidiasis
Source: J Nanobiotechnology. 2023 Feb 13;21:53. doi: 10.1186/s12951-023-01800-x (PMC9926847; doi:10.1186/s12951-023-01800-x)
Supplement: Supplementary file 1 — Additional file 1: Fig. S1. The viability effect of ultrasound on macrophages A and L. crispatus B treated at intensity of 0.5 W/cm2, 1.0 W/cm2 and irradiation time of 5 min, 10 min, 15 min in 50% duty cycle of pulse mode. Fig. S2. The comparison of macrophages viability A and L. crispatus viability B treated with free AmB and AmB-NPs at different concentrations of AmB from 0 to 16 μg/mL. Fig. S3 Observation of constructed vulvovaginal candidiasis model in rabbits after 3 days and 5 days of continuous injection of C. albicans solution into the vagina by Macroscopical, Gram staining (bar = 10 μm), HE staining (bar = 100 μm) and C. albicans culture of vaginal lavage fluid on SDA. Fig. S4. H&E stained images of rabbit vaginas conducted by different treatments (scale bar = 20 μm, 40 × magnification). Fig. S5. PAS stained images of rabbit vaginas conducted by different treatments (scale bar = 20 μm, 40 × magnification). [file 12951_2023_1800_MOESM1_ESM.docx]

## Additional file 1

## Low Intensity Ultrasound-Mediated Drug-Loaded Nanoparticles Intravaginal Drug Delivery: An Effective Synergistic Therapy Scheme for Treatment of Vulvovaginal Candidiasis

Min Yang^1,2^, Yuchao Cao^1,2^, Zhifei Zhang^1,2^, Jiajun Guo^1,2^, Can Hu^1,2^, ZhibiaoWang^1,2^*, YonghongDu^1,2^*

^1^State Key Laboratory of Ultrasound in Medicine and Engineering, College of Biomedical Engineering, Chongqing Medical University,Chongqing, 400016, China.

^2^Chongqing Key Laboratory of Biomedical Engineering, Chongqing Medical University, Chongqing, 400016, China.

Correspondence to: Yonghong Du, No. 1 Yixueyuan Road, Yuzhong District, Chongqing 400016, China.

Correspondence to: Zhibiao Wang, No. 1 Yixueyuan Road, Yuzhong District, Chongqing 400016, China.

1. mail addresses: duyonghong@cqmu.edu.cn (Y. Du); wangzhibiao@cqmu.edu.cn (Z.Wang).

**1.** **Effect of ultrasound on macrophages and Lactobacillus** **activity**

Macrophages and Lactobacillus were sonicate with different ultrasonic parameters respectively to selected the safe dose of ultrasound irradiation. The activity of macrophages decreased with the increase of ultrasonic intensity and irradiation time, and the cellular activity was higher than 80% under ultrasonic irradiation at intensity of 0.5 W/cm^2^,1.0 W/cm^2^ for 5 min. However, the cell activity decreased significantly after the irradiation time was extended to 10min and 15min, although at the lowest intensity of 0.5 W/cm^2^ (Fig. S1A). Conversely, ultrasound irradiation can promote the proliferation of *L. Crispatus* under the low intensities of 0.5 W/cm^2^, 1.0 W/cm^2^ for 5min and 10min, as shown by a 1.9-fold increase in cell activity after 5 min of 1.0 W/cm^2^ irradiation compared to the controls (Fig. S1B). However, the bacterial activity decreased sharply when the irradiation time was extended to 15 min.


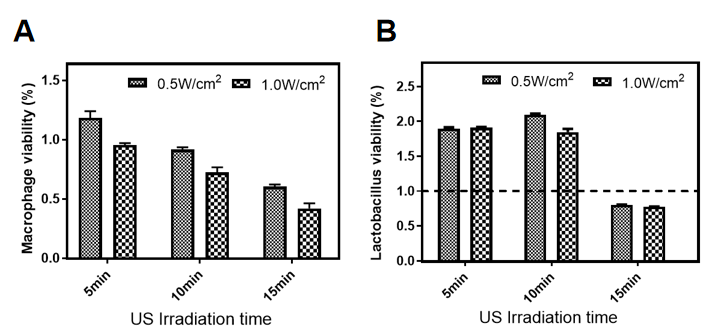


**Fig. S1.** The viability effect of ultrasound on macrophages (A) and *L. crispatus* (B) treated at intensity of 0.5 W/cm^2^, 1.0 W/cm^2^ and irradiation time of 5 min, 10 min, 15 min in 50% duty cycle of pulse mode.

**2. Cytotoxicity of AmB-NPs on macrophages and Lactobacillus**

The cytotoxicity of AmB-NPs relative to free AmB against normal macrophages RAW264.7 and *L. crispatus* in vitro and the biosafety of AmB-NPs in vivo were examined as shown in **Fig.S2**. The activity of macrophages decreased with the increase of AmB concentration and the activity was significantly decreased to 72% compared to the controls at the concentration of 4.0 μg/mL, while the cell activity in AmB-NPs group was significantly higher than that of free AmB at the same drug concentration (P < 0.01) and that was still about 80% at the concentration of 16 μg/mL **(Fig. S2A)**. Similarly, the activity of *L. crispatus* was not significantly reduced at equivalent AmB concentrations of 16 μg/mL in AmB-NPs but that was reduced to 60% with the co-culture of free AmB **(Fig. S2B)**. RAW264.7 macrophages were co-cultured with AmB-NPs and free AmB at varying equivalent AmB concentrations (1.0, 2.0, 4.0, 8.0, 16.0 μg/mL)


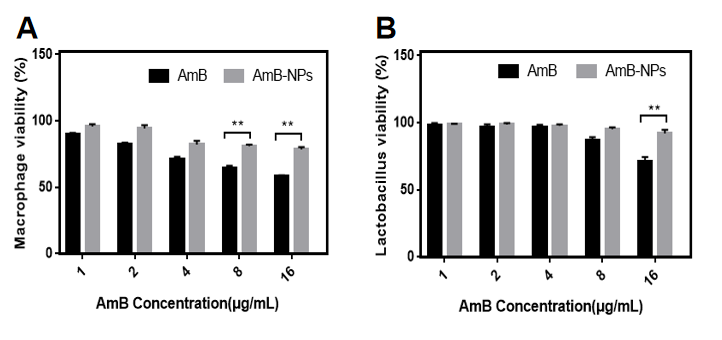


**Fig. S2.** The comparison of macrophages viability (A) and *L. crispatus* viability (B) treated with free AmB and AmB-NPs at different concentrations of AmB from 0 to 16 μg/mL.

**3. Effect of ultrasound on macrophages and Lactobacillus activity**

The constructed VVC model in rabbits after 3 days and 5 days of continuous injection of *C. albicans* solution into the vagina were observed by Macroscopical, Gram staining, HE staining and *C. albicans* culture of vaginal lavage fluid on SDA, as shown in Fig.S3. The VVC model was successfully established VVC model of rabbits was successful established on the 5th day after continuous injection of *C. albicans* solution into the vagina by observing a red, swollen of the vulva, a large number of *C. albicans* and hyphae under Gran staining, and invasion colonizing of *C. albicans* hyphae on the mucosal surface of the vagina and aggregation of inflammatory cells in the submucosa under HE staining. Moreover, vaginal lavage fluid cultured on SDA showed extensive *C. albicans* fungal colony growth.


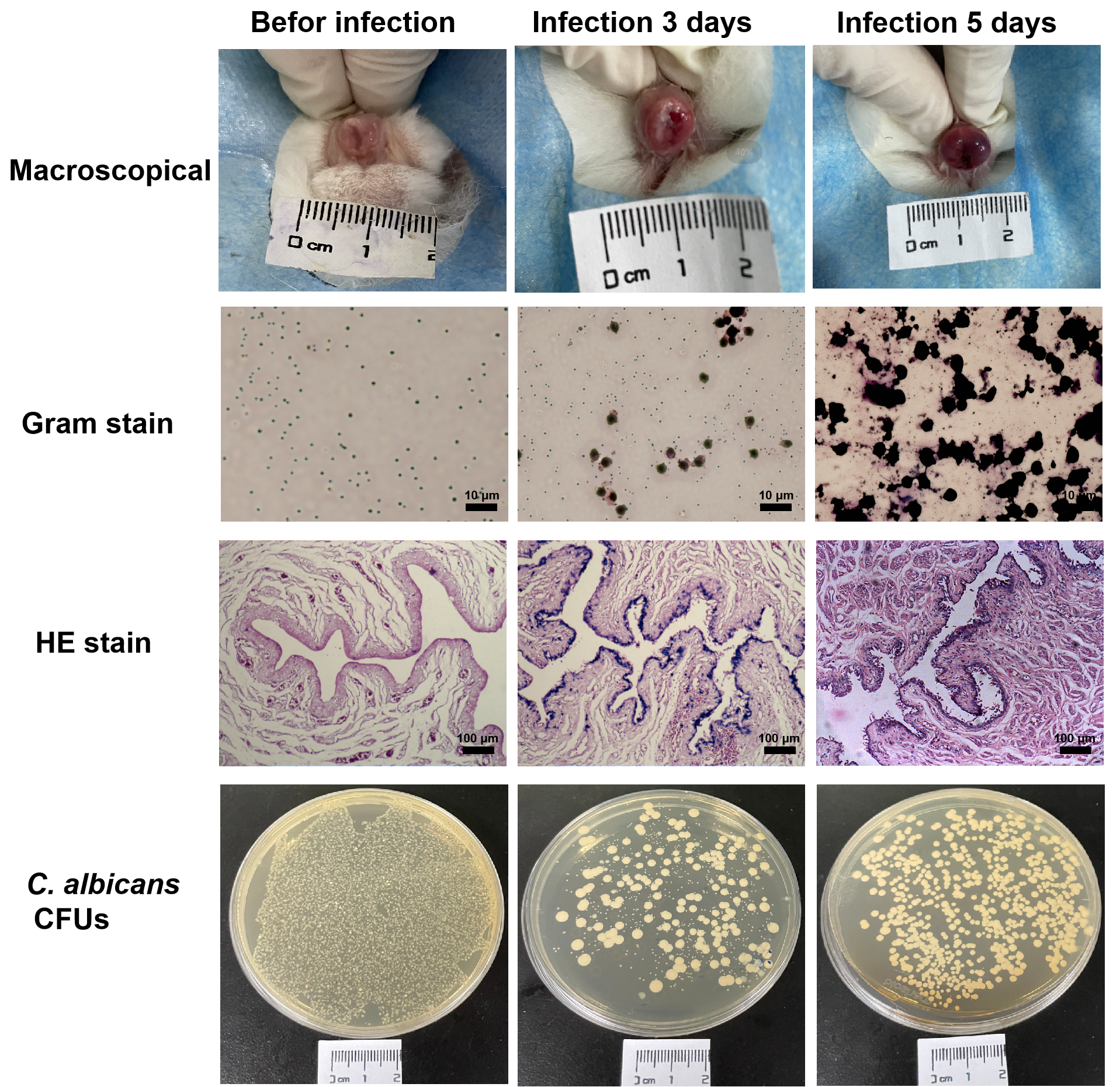


**Fig. S3** Observation of constructed vulvovaginal candidiasis model in rabbits after 3 days and 5 days of continuous injection of *C. albicans* solution into the vagina by Macroscopical, Gram staining (bar = 10 μm), HE staining (bar = 100 μm) and *C. albicans* culture of vaginal lavage fluid on SDA.


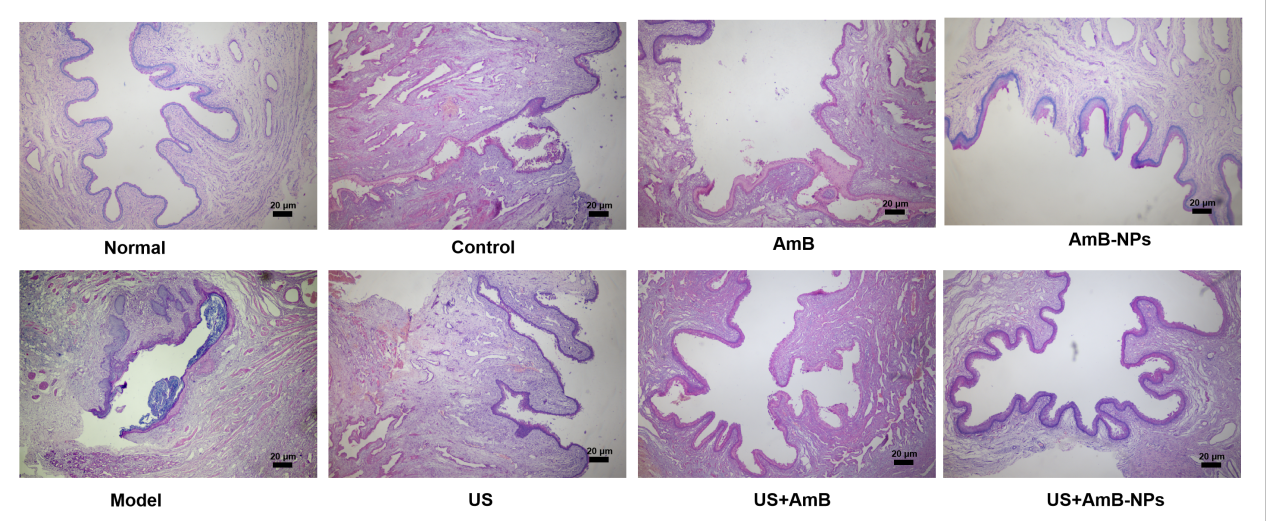


**Fig S4.** H&E stained images of rabbit vaginas conducted by different treatments (scale bar = 20 μm, 40x magnification).


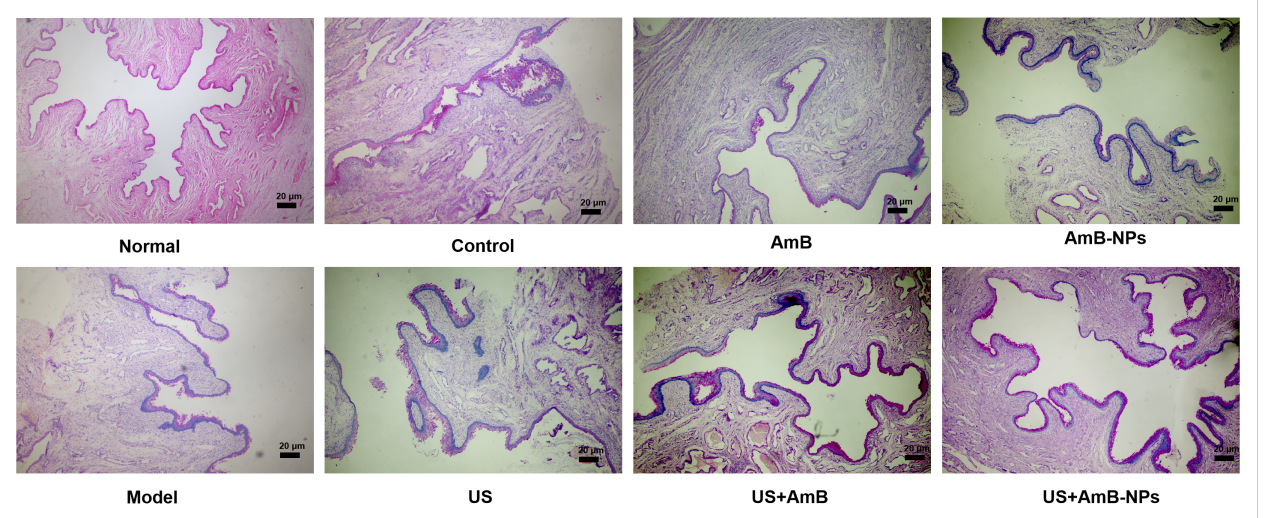


**Fig. S5.** PAS stained images of rabbit vaginas conducted by different treatments (scale bar = 20 μm, 40x magnification).
